# Investigating transcriptome-wide sex dimorphism by multi-level analysis of single-cell RNA sequencing data in ten mouse cell types

**DOI:** 10.1186/s13293-020-00335-2

**Published:** 2020-11-05

**Authors:** Tianyuan Lu, Jessica C. Mar

**Affiliations:** 1 Australian Institute for Bioengineering and Nanotechnology, The University of Queensland, Brisbane, QLD 4072 Australia; 2 Quantitative Life Sciences Program, McGill University, Montreal, QC H3A 0G4 Canada

**Keywords:** Single cell RNA sequencing, Sex dimorphism, Differential expression, Differential distribution, Cell cluster, Transcription regulatory network, Pathway analysis

## Abstract

**Background:**

It is a long established fact that sex is an important factor that influences the transcriptional regulatory processes of an organism. However, understanding sex-based differences in gene expression has been limited because existing studies typically sequence and analyze bulk tissue from female or male individuals. Such analyses average cell-specific gene expression levels where cell-to-cell variation can easily be concealed. We therefore sought to utilize data generated by the rapidly developing single cell RNA sequencing (scRNA-seq) technology to explore sex dimorphism and its functional consequences at the single cell level.

**Methods:**

Our study included scRNA-seq data of ten well-defined cell types from the brain and heart of female and male young adult mice in the publicly available tissue atlas dataset, Tabula Muris. We combined standard differential expression analysis with the identification of differential distributions in single cell transcriptomes to test for sex-based gene expression differences in each cell type. The marker genes that had sex-specific inter-cellular changes in gene expression formed the basis for further characterization of the cellular functions that were differentially regulated between the female and male cells. We also inferred activities of transcription factor-driven gene regulatory networks by leveraging knowledge of multidimensional protein-to-genome and protein-to-protein interactions and analyzed pathways that were potential modulators of sex differentiation and dimorphism.

**Results:**

For each cell type in this study, we identified marker genes with significantly different mean expression levels or inter-cellular distribution characteristics between female and male cells. These marker genes were enriched in pathways that were closely related to the biological functions of each cell type. We also identified sub-cell types that possibly carry out distinct biological functions that displayed discrepancies between female and male cells. Additionally, we found that while genes under differential transcriptional regulation exhibited strong cell type specificity, six core transcription factor families responsible for most sex-dimorphic transcriptional regulation activities were conserved across the cell types, including ASCL2, EGR, GABPA, KLF/SP, RXRα, and ZF.

**Conclusions:**

We explored novel gene expression-based biomarkers, functional cell group compositions, and transcriptional regulatory networks associated with sex dimorphism with a novel computational pipeline. Our findings indicated that sex dimorphism might be widespread across the transcriptomes of cell types, cell type-specific, and impactful for regulating cellular activities.

**Supplementary information:**

**Supplementary information** accompanies this paper at 10.1186/s13293-020-00335-2.

## Background

The fundamental functional unit of all living organisms is a single cell. While cells of the same organism share largely identical genetic material, numerous mechanisms regulate the genome to give rise to diverse cell type-specific behaviors. It has long been recognized that the specificity of cell types and the tissues from which they are derived from, are driven by transcriptional regulatory programs that lead to distinct patterns of gene expression. For instance, one gene may have different mean expression levels in different cell types or may be expressed by different fractions of cells in different tissues [1]. Existing studies have shown that even in isogenic cells, cells from the same cell type or identity, gene expression is a stochastic and heterogeneous process that is fundamental to controlling cell fate [1–3]. As a consequence, the variable yet well-organized transcriptome not only ensures normal growth and functioning of each individual cell throughout its lifetime, but also enables cooperation between cells to function as a multi-cellular entity that make up a living organism.

Sex is widely associated with many crucial phenotypic differences and is also linked to various transcriptional variabilities. For instance, in mice, a considerable fraction of genes (from 14% in the brain to 70% in the liver) are significantly differentially expressed in various tissues between females and males [4]. Genes exhibiting sex-dimorphic expression levels are enriched in biologically important pathways, including immune response and lipid metabolism in the liver, amine metabolism in muscle, and ATPase activity in the brain [4]. Several hormone response-related transcription factors, such as zinc finger proteins (ZFPs) and STAT proteins in the liver [5, 6], that globally regulate gene expression modules have also been shown to be involved in sex-dimorphic regulatory mechanisms. However, to this end, most studies investigating sex-dimorphic gene expression and transcriptional regulation are based on bulk sample RNA sequencing or microarrays, where RNA transcripts from a large number of cells are amalgamated and the averaged gene expression levels are derived. Though informative in various contexts, such analyses conceal cell-to-cell variation that may be important in characterizing differences between cell types present in the same sample. Conceivably, if two cell types of similar proportions both differentially express the same gene but in opposite directions between females and males, comparisons that average cell type-specific gene expression levels may overlook such differences. This limitation may introduce more bias in studying heterogeneous tissues and organs, such as the brain and heart. Accurately accounting for cellular heterogeneity or isolating and separately analyzing different types of cells by flow cytometry are certainly promising to resolve these issues. For instance, a recent study identified 3161 genes displaying sex-biased gene expression in placental mammals after adjusting for confounding effects associated with cell type proportions [7]. Another study applying flow cytometry revealed that 144 genes are differentially expressed between human males and females in immune cells, among which 75% are located on autosomes [8]. These findings substantially enriched characterization of sex dimorphism in the transcriptome. However, information of tissue structure and intracellular distributions are unlikely to be preserved in bulk sample data analysis, whereas a large sample size in flow cytometry studies might not be easily achieved due to the possibly high costs incurred and indispensable facility requirements.

The application of single cell RNA sequencing (scRNA-seq) technology to generate high-throughput data on a per-cell basis has thus advanced our understanding of transcription regulation by elucidating how biological variability is controlled through gene expression. Most scRNA-seq analyses focus on two levels. First, at the cellular level, cluster analysis and cell type annotation are important for understanding the representation of different cell identities in a sample [9]. Trajectory inference methods, such as Monocle, also pioneered the possibility of assigning cell-cell ordering and present an overall model that allowed for the inference of gene expression dynamics [10]. Second, at the gene level, many earlier studies followed common practices in the analysis of differential expression, construction of regulatory networks, and gene set enrichment analysis that have been explored and adapted from bulk RNA-seq data.

It is important to highlight that the merit of scRNA-seq data is never limited to inferences based on the average expression level. While differentiation among homogeneous cells may be identified by models that permit bi-modality or even multi-modality, variance and higher moments of the distribution of transcript read counts can provide deeper insights into how subgroups of cells are transcriptionally regulated [11]. For instance, changes in gene expression variability may imply a shift in the regulatory dynamics between two phenotypes. Modeling multi-modality of a gene expression distribution may be an informative characteristic of scRNA-seq data as it allows for the identification of a mixture of cells in different states. Furthermore, models that incorporate more flexible representations of variability through the commonly over-dispersed variance in parameterized negative binomial model are also approaches that have been underexplored. Despite some efforts to build knowledge of multi-modality into cell type clustering algorithms [12] and the statistical identification of multi-modality in scRNA-seq data [13], the importance of modeling gene expression variability and its impact on understanding biological regulation has been inadequately addressed.

In this study, we make effective use of scRNA-seq data from a recently released compendium, Tabula Muris, to decode sex dimorphism-associated transcriptional variability from different perspectives mentioned above using a novel computational pipeline. Given extensive studies that have focused on the biology of sex differentiation and dimorphism, we illustrate how scRNA-seq can confirm previous findings based on bulk RNA-seq and provide new insights into sex dimorphism in terms of gene expression variability, cell composition, and the regulation of transcriptional networks in the brain and heart.

## Methods

### Data source and preprocessing

With a large volume of data and high sequencing quality compared to similar studies, the Tabula Muris study presented scRNA-seq data of 20 organs collected from a total of four young male and three virgin female C57BL/6JN mice that were controlled for age, environment, and epigenetic effects [14]. Samples of each organ were collected from the same anatomical regions [14]. Fluorescence-activated cell sorting (FACS)-processed scRNA-seq data were downloaded from the Tabula Muris study (https://github.com/czbiohub/tabula-muris, accessed July 15, 2018) and our study chose to focus on cell types derived from the brain and heart. In order to ensure adequate statistical power in downstream analyses, we selected the four types of brain cells and six types of heart cells that met sufficient sample size requirements (scRNA-seq data for 50 or more cells) in both female and male individuals. The set of ten cell types were astrocytes of the cerebral cortex, brain endothelial cells, microglial cells, oligodendrocytes, cardiac muscle cells, endocardial cells, heart endothelial cells, cardiac fibroblast cells, and leukocytes sampled from heart and cardiac smooth muscle cells (see Supplementary Table 1).

Following the same criteria adopted by the Tabula Muris study [14], we excluded cells of low sequencing quality as these would affect the performance of downstream imputation methods to correct for technical dropouts. Specifically, we required that each cell should have ≥ 500 genes with at least one read count and that each cell should have ≥ 50,000 total read counts after subtracting counts for Rn45s, the 45S pre-ribosomal RNA that is uniformly overabundant [14]. We simultaneously excluded genes that were deemed undetected or on the Y chromosome. Moderately detected genes were defined as having a read count ≥ 5 in a cell. Genes that were moderately detected in ≥ 5 cells were retained for further analysis.

We imposed the two preliminary filters above on all brain cells and all heart cells, respectively, and obtained 6498 brain cells with 15,268 detected genes as well as 4186 heart cells with 15,160 detected genes.

### Imputation and validation

Zero inflation (excessive zero read counts) arising from the low RNA capture rate in scRNA-seq data is a universal obstacle [9]. Namely, a dropout occurs where one gene is moderately expressed in some cells but is not detected due to a technical failure to capture enough transcripts. This type of zero read count is distinct in nature from the case where a transcript may be undetectable because it is either suppressed or not required in the gene expression program in a cell. It is essential to distinguish the technical dropouts from true instances where a transcript has not been expressed, both cases which lead to numerical zero read counts. Since expression variability plays an important role in this study, we imputed the filtered data by scImpute [15] to prevent the overrepresentation of variance. This method first clusters cells into subpopulations using semi-unsupervised spectral clustering with the number of intended subpopulations specified. It then fits a normalized expression distribution for each gene in each subpopulation using a gamma-normal mixture model. Imputation is performed cell by cell, where read counts of zero after imputation ideally represent only events where no transcript is made, and the gene is considered silenced.

We used the R package scImpute with dropout threshold of 0.5 to impute the filtered brain and heart count matrices separately, including both female and male cells. The dropout threshold setting has been shown to be inconsequential by Li and Li [15]. The number of cell clusters was set to four for brain cells and six for heart cells, reflecting the number of cell types selected for each organ. Dropout values in the output read count matrices were estimated cell by cell, and the imputed values were not normalized.

For each gene, negative binomial distributions were fitted based on the imputed and original read counts, respectively. Negative binomial likelihood was obtained and compared to test whether the imputation refined characterization of distributional properties.

### Normalization

Counts in the imputed matrices were further log-normalized while accounting for batch effects based on detection of high-dimensional mutual nearest neighbors [16]. Normalization and batch effect removal was performed using the R package Seurat and SeuratWrappers with default settings of the “LogNormalize” and “RunFastMNN” functions [16, 17].

### Validation of clustering

We selected highly variable genes (HVGs) to validate clustering of cells done by the Tabula Muris dataset and examine what effect imputation had on the results. We selected genes with standardized log dispersion > 0.5, and with expression mean > 0.0125 and < 3 as HVGs. These parameters were chosen to be consistent with the Tabula Muris study. Expression dispersion and mean were calculated by Seurat. We then visualized the brain and cells separately using a 2-dimensional t-distributed stochastic neighbor embedding (tSNE) map after projecting the normalized data onto the first 50 principal components (PCs). tSNE was done by the R package Rtsne with a perplexity of 30 for five times using five different random seeds. Assessing whether a cell was grouped into its identified cell type was based on the nearest neighbor principle (Supplementary Figure 1). Eight brain cells and 24 heart cells (Supplementary Table 1) were removed due to recurrent inconsistent grouping with their identified cell type, i.e., their nearest neighbors on the two-dimensional tSNE map were annotated to a different cell type. To examine whether the imputation introduced spurious sex dimorphism, we compared the distributions of library sizes between female and male cells for each type of cell.

### Differential distribution analysis

We implemented scDD to identify differential distributions of genes on the imputed and normalized data, in brain and heart cells, respectively. scDD aims to classify differentially distributed genes into several categories: differential expression (DE), differential modality (DM, where gene expression is unimodal in one condition versus bi-modal in the other condition) with one overlapping component, differential proportion (DP) of cells within each component, differential modality without an overlapping component (differential both, DB), and more complex scenarios that cannot be categorized (NC). Differential distributions were tested using the Kolmogorov-Smirnov test. We obtained these differentially distributed genes with a Bonferroni-Hochberg corrected *p* value (false discovery rate; FDR) < 0.05. In particular, we kept the FDR threshold comparably strict across different cell types. Therefore, we further required DE genes should have FDR among the smallest 3% in all genes under investigation in each cell type. This threshold was chosen as it made the highest FDR cutoff at around 0.05 in the cell type of the least sample size while lower FDR cutoff in cell types of larger sample sizes. DE genes should also have an absolute difference over 0.2 between female and male in normalized log_10_-mean expression values. This difference corresponded to (10^0.2^≈) 1.5-fold change in read counts. scDD also assessed a gene’s differential proportion of zeros (DZ) by performing logistic regression between two groups. Genes with a *χ*^2^ test FDR < 0.025 were categorized as DZ genes. Venn diagrams visualizing overlaps were plotted using jvenn [18].

### Gene set variation analysis

To illustrate how differentially regulated genes can influence various processes and how cells exhibit heterogeneity, we performed gene set variation analysis (GSVA) of the differential distribution (DD) genes using the R package GSVA [19]. GSVA outputs an enrichment score for each cell; thus, cells can be directly compared as opposed to other pathway-based methods that only compare male and female groups directly. 4269 Gene Ontology (GO)-derived gene sets of high quality were retrieved online (http://www.go2msig.org/cgi-bin/prebuilt.cgi?taxid=10090, accessed September 30, 2018). GSVA generates enrichment scores of every gene set for every cell: positive scores indicate increased pathway activity that is inferred from relative overall gene expression levels assessed by a Kolmogorov-Smirnov-like random walk statistic, while negative scores indicate weakened pathway activity. Expression matrices of DD genes in each cell type were supplied to GSVA and all default parameters were used. We performed Welch’s *t* tests and identified pathways that were significantly differentially represented between female and male groups using an FDR < 5 × 10^−5^ and an absolute GSVA score difference > 0.1.

For four types of cells with > 100 significantly differentially represented gene sets, we visualized keywords of the gene sets represented using the R package wordcloud. Common non-specific descriptive words were removed from the gene set names, including “regulation”, “activity”, “process”, “activity”, “cell”, “response”, “positive”, and “negative”.

### Identification of sub-cell types

The R package Seurat was used to perform unsupervised clustering of 2033 heart fibroblast cells. We retrieved imputed but not normalized gene expression matrix of all 3428 DD genes of these cells, normalized this matrix de novo using the “LogNormalize” function and identified 775 HVGs (also with standardized log dispersion > 0.5, and with expression mean > 0.0125 and < 3) as potential classifiers. We then decomposed the correlation structure using principal component analysis (PCA) and fed the first nine PCs into the built-in “FindClusters” function of Seurat, which implements a shared nearest neighbor modularity optimization-based clustering algorithm. The first nine PCs were PCs explaining > 2% of the total variance each. The parameter “resolution” was set to 0.3, which controls the number of clusters. All other default parameters were used. Clusters were visualized using tSNE after projecting the normalized data onto the first nine PCs.

For each of the five clusters identified, we first identified marker genes that distinguished the cluster from the other four clusters using the built-in “FindAllMarkers” function, requiring ≥ 25% genes to be expressed in either of the two populations (i.e., the cluster being tested and the other four clusters as an entity) and leaving other settings as default. Ten marker genes (with the smallest FDR) of each cluster were gathered and used for visualizing sub-cell type-specific gene expression. We further identified marker genes that distinguished clusters (0 and 1; 2 and 3) with the same dominating sex, using the “FindMarkers” function with default settings. Significant marker genes had an FDR < 0.05.

To examine the validity of these clusters, we also investigated whether housekeeping genes and cell division or mitotic cell cycle-related genes were differentially expressed between female and male cells. We retrieved 27 consistently expressed mouse housekeeping genes [20] and tested for differential gene expression between female and male cells in these identified sub-clusters. We also obtained 588 genes associated with cell division as well as 833 genes associated with mitotic cell cycle in mouse from the Mouse Genome Informatics [21]. We performed gene set enrichment analysis using the R package fgsea (http://bioconductor.org/packages/release/bioc/html/fgsea.html) for each cell and compared the distribution of normalized enrichment scores between female and male cells in each identified sub-cluster.

### Gene regulatory network construction by Passing Attributes between Networks for Data Assimilation (PANDA)

By studying DE genes and DD genes, we focused on uncovering what effects two different modes of differentially regulated genes have. Yet the question remains as to whether drivers of gene regulation show sex dimorphism. Transcription factors (TFs) are undoubtedly one of the most important drivers of transcriptional regulation. Hence, we studied gene regulatory networks driven by TFs using PANDA [22], which has been successfully implemented for bulk RNA-seq data. PANDA exhaustively utilizes information of co-expressed genes where TF-gene interactions are inferred by the detection of transcription binding motifs in the promoter and a priori inter-TF protein-protein interaction network. PANDA evaluates the strength of the interaction marked by each edge connecting one TF and one targeted gene. We augmented this approach to identify subsets of the TF networks that were sex-specific by comparing the edges in networks that have been established in different types of cells between female and male.

#### Transcription factor motif

We downloaded the GRCm38 (mm10) genome assembly of *Mus musculus* and its annotations of transcription start sites (TSSs) from USCS Genome Browser (https://genome.ucsc.edu/cgi-bin/hgTables, accessed August 10, 2018). We retained genes with one or two annotated TSSs. Genes with more than two TSSs were discarded because they may represent more complex regulatory mechanisms that cannot be explicitly explained in this study. We defined the promoter as a (− 750, + 250) region (in the unit of base pair) around the TSS of single-TSS genes and as a (− 1000, + 500) region around the middle point of two TSSs for double-TSS genes. We also downloaded position weight matrices (PWM) of 663 *Mus musculus* transcription factor (TF) motifs available at the Catalog of Inferred Sequence Binding Preferences (http://cisbp.ccbr.utoronto.ca/, accessed August 5, 2018). For each combination of a TF and the promoter region of a gene, we examined the potential existence of TF-binding by mapping the PWM of the TF to the promoter region using the Find Individual Motif Occurrences software. We obtained potential TF-promoter binding pairs between 359 TFs and 14,851 genes with a *p* value threshold of 5 × 10^−5^.

#### Protein-protein interaction

We obtained a subset of the protein-protein interaction network covering all 359 TFs identified from step 1 of the PANDA network analysis. Interaction scores were retrieved from StringDb version (v.) 10 (https://string-db.org, accessed August 15, 2018).

#### PANDA networks

We first removed genes on sex chromosomes for both brain and heart cells and retained genes which passed quality control during preprocessing and had a potential active TF-binding site. As a result, we obtained 9673 genes regulated by 318 TFs in 6490 brain cells as well as 9722 genes regulated by 317 TFs in 4162 heart cells. Specifically, TFs on the sex chromosomes were not discarded (e.g., AR on the chromosome X). TF-motif regulatory matrix, protein-protein interaction matrix, and read count matrix were trimmed accordingly. Finally, a gene was used for network construction if it (i) was a moderately expressed autosomal gene, (ii) had one or two annotated TSSs, and (iii) could potentially interact with at least one TF identified. For each cell type, we adopted a previously established Jack-knife method to construct sex-specific regulatory network ensembles [23]. We randomly selected 10 cells of the same sex to construct one PANDA regulatory network and used the PANDA program to incorporate the information collected above. We repeated this random selection and network construction procedure until we obtained 100 female-specific and 100 male-specific networks for each cell type.

#### Identification of differentially represented edges

The *Z*-scores generated by PANDA indicate the regulatory effectiveness of TFs and are normally distributed. We performed Welch’s *t* tests to compare the resulting *Z*-scores between female and male cells to investigate sexually dimorphic targets for every cell type. Edges of negative mean scores in both female and male network ensembles were discarded as they were identified as non-existent regulatory relationships. We obtained differentially represented edges with an absolute *Z*-score difference > 0.25 and an FDR < 5 × 10^−5^.

#### Identification of differentially targeting TFs

The overall activity of each TF was measured by the “out-degree” score, originally defined by Glass et al. [23]. In each sex-specific network, the edge weights of active edges (i.e., edges of positive mean scores in at least one ensemble) involving one specific TF were summed. Hence, we obtained 100 female-specific and 100 male-specific out-degree scores for every TF. We performed Welch’s *t* tests to compare the out-degrees of TFs in female-specific and male-specific ensembles and defined TFs with an absolute mean out-degree difference > 10 and an FDR < 0.05 as differentially targeting TFs.

#### Identification of differentially targeted genes

Similarly, we measured the extent to which each gene is under the regulation of TFs by “in-degree” scores [23]. In each sex-specific network, the edge weights of active edges involving one specific gene were summed. We also performed Welch’s *t* tests and defined differentially targeted genes as those having an absolute mean in-degree difference > 10 and an FDR < 0.05.

### Functional enrichment analysis

GO annotations of *Mus musculus* genes were retrieved from the Mouse Gene Informatics database (http://www.informatics.jax.org/function.shtml, accessed August 15, 2018). The R package topGO was used for GO term enrichment analysis with the default settings. Statistical significance was set at *p* value < 0.05 and an overlap of ≥ 5 genes with the background set. Since the tests performed by topGO were not considered independent, no correction for multiple-testing was applied and instead, a ranked list of GO terms was considered [24]. When performing GO term enrichment analyses of DE genes and marker genes of sub-cell types, we set the background gene list as the 15,268 genes and 15,160 genes that passed preliminary quality control for brain cell types and heart cell types, respectively. Similarly, when performing GO term enrichment analyses of genes on differentially represented edges or genes that were overall differentially targeted, we set the background gene list as 9673 genes and 9722 genes that were used for network construction for brain cell types and heart cell types, respectively.

Pathway analysis was performed using the R package clusterProfiler with the Kyoto Encyclopedia of Genes and Genomes (KEGG) as pathway definitions. KEGG annotations of *Mus musculus* genes were retrieved from the R database org.Mm.eg.db. Statistical significance was set at FDR < 0.1 and with an overlap of ≥ 5 annotated genes in the pathway. Pathways were visualized using the R package pathway.

## Results

### A computational pipeline to identify gene regulatory networks for multiple cell types from single cell RNA sequencing data to understand sex-specific differences in gene expression

**Fig. 1:**
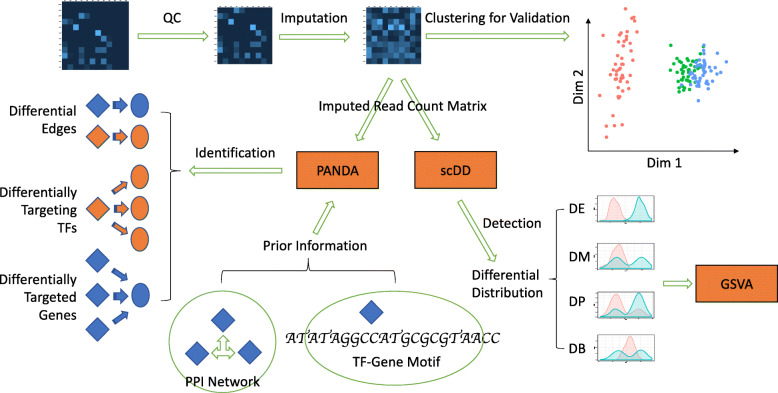
Schematic overview of processing cell type-specific scRNA-seq data. Expression data were cleaned, imputed, and validated by clustering. Differential distribution analysis using scDD identified differentially expressed genes and various forms of differential distributions. Differentially distributed genes were fed into GSVA to illustrate differential representation of pathways in each individual cell. Gene co-expression information was combined with information of TF-gene motifs and TF-TF interactions in PANDA network inference. Differentially activated TF-gene couplings (edges), differentially active TFs, and genes under differential intensity of TF-regulation were studied and compared among different cell types. TFs are represented by diamonds. Genes are represented by ovals

This study set out to investigate heterogeneity in the transcriptional regulation of sex-specific differences in tissues from the Tabula Muris dataset. A computational pipeline was developed to process the single cell RNA sequencing (scRNA-seq) data and identify gene regulatory networks that were tissue type and sex-specific (Fig. 1). Our study was built from ten cell types from the brain and heart samples in Tabula Muris [14] and restrictions to these ten cell types was motivated by the availability of adequate sample size of the data. Imputation of the data did not introduce spurious sex dimorphism as the library sizes after imputation increased proportionately for both female and male cells (Supplementary Figure 2). On the other hand, the overall data quality significantly improved as the imputed data better reflected characteristics of the negative binomial distribution (Supplementary Figure 3). While validating our imputed and normalized data, we found that only 0.12% of the brain cells and 0.57% of the heart cells (Supplementary Figure 1 and Supplementary Table 1) showed inconsistent grouping with the original cell type that they were annotated to. These cells were removed from subsequent analyses. The dominant source of variability in gene expression was driven by cell type differences and, overall, the cells did not show clear separation with respect to sex (Supplementary Figure 1). No evidence of mouse sample-driven gene expression pattern was observed (Supplementary Figure 1).

### Identifying genes with changes in differential distribution versus differential expression between male and female mice to characterize sex dimorphism

We investigated sex-specific differential distribution of gene expression separately in each cell type using scDD [13]. For each gene, scDD first models the log-transformed nonzero expression values using a conjugate Dirichlet process mixture of normal distributions, which partitions the expression values and identifies the modality of a gene’s expression distribution. scDD categorizes differential distributions occurring between two groups as one of the following categories DE, DM, DP, DB, and NC (the “Methods” section and Supplementary Figure 4). In addition, logistic regression models can be used to assess whether genes showed a differential proportion of zeros between female and male groups after adjusting for the proportion of actively expressed genes in each cell.

In this study, significant DE genes classified by scDD were viewed as a separate category, since these genes represent differential regulation of gene expression in a standard sense. In contrast, the genes classified into the DM, DP, DB, DZ, and NC categories were the main focus of this study because of their altered non-standard distributions in the scRNA-seq profiles between males and females. We designated the union of genes classified as DM, DP, DB, DZ, and NC as DD genes and provide summary statistics for all genes in Supplementary Table 2. No significant sex-dimorphic expression of housekeeping genes, such as eukaryotic translation initiation factors and proteasome subunits (Supplementary Table 2), was observed.

### Differentially expressed genes exhibit high cell type specificity

**Fig. 2:**
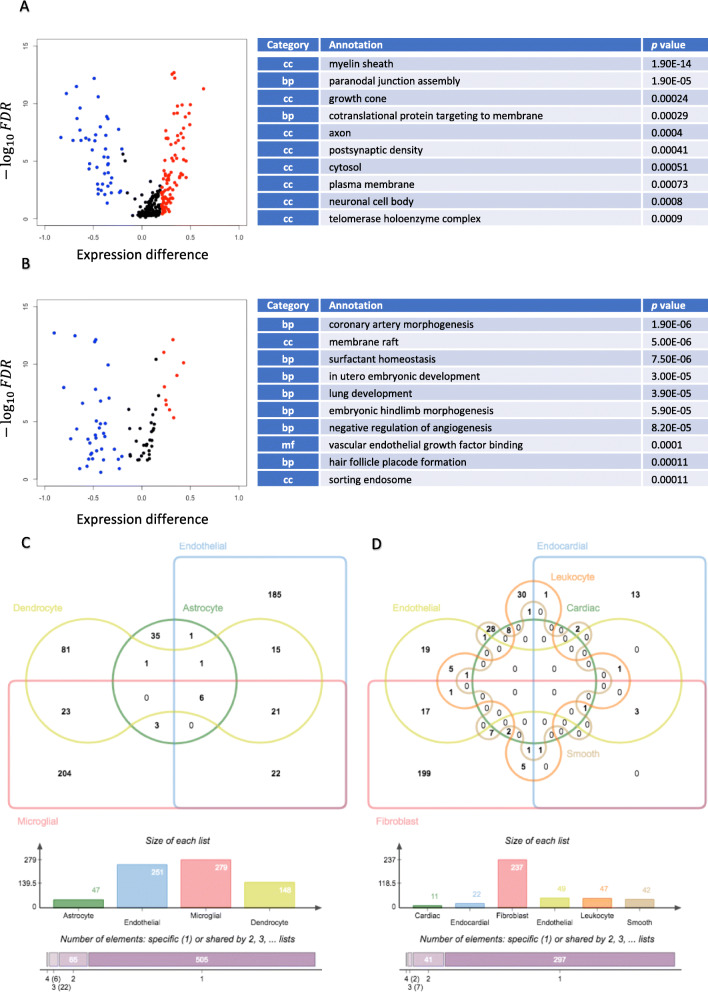
Cell type-specific differential gene expression. Volcano plots and GO annotations of differentially expressed genes in **a** oligodendrocytes and **b** heart endothelial cells exhibited cell type-specific gene expression and related pathways. Distribution of DE genes showed disequilibrium in females (red) and males (blue). In oligodendrocytes, more genes had female-specific expression, whereas in heart endothelial cells more had male-specific expression. For each cell type, only genes identified by scDD as DE genes having an FDR among the smallest 3% were retained. Only ten enriched GO terms of the smallest *p* values were presented. GO terms were categorized into biological process (bp), cellular component (cc), and molecular function (mf). Venn diagrams of **c** brain cells and **d** heart cells showed imbalanced number and low conservation of DEGs across cell types. Bar graphs record number of DEG in each cell type. In brain cells, 505 DEGs were specific to one cell type, while 65, 22, and 6 DEGs were shared by two, three, and four cell types; in heart cells, 297 DEGs were specific to one cell type, while 41, 7, and 2 DEGs were shared by two, three, and four cell types. “Dendrocyte” in **a** represents oligodendrocyte, “Cardiac” in **b** represents cardiac muscle cell, and “Smooth” in **b** represents smooth muscle cell

Significant DE genes between male and female mice were detected in all four brain cell types and all six heart cell types. These genes had unimodal distributions with different mean expression levels between sexes and were associated with biological processes that relate to tissue-specific regulation (Fig. 2a, b; Supplementary Table 3).

It is interesting to note that all cell types exhibited sex-specific differential expression in ribosome subunits. This result may serve as the foundation for a wide variety of differences in intracellular biochemical reactions by influencing the overall regulation of translation. Thirty-two genes coding for large ribosomal subunits and 31 genes coding for small ribosomal subunits had sex-biased expression in at least one cell type in this study, among which *Rps25* was more highly expressed in female astrocytes, brain endothelial cells, microglia, oligodendrocytes, cardiac fibroblasts, cardiac smooth muscle cells, and cardiac leukocytes. Similarly, *Rpl6*, was more highly expressed in female brain endothelial cells, microglia, endocardial cells, cardiac leukocytes.

While most genes that were differentially expressed between the sexes were not shared between cell types some notable exceptions were observed (Fig. 2c, d). For example, the mitochondrial leucyl-tRNA synthetase, *Lars2*, was differentially expressed in all four brain cell types and four other heart cell types where males had significantly higher *Lars2* expression, which could contribute to sex-dimorphic mitochondrial functions in multiple cell types. Long non-coding RNA *Malat1* was more highly expressed in male endocardial cells, fibroblasts, heart leukocytes, and heart smooth muscle cells, suggesting widespread sex-dimorphic post-transcriptional regulation [25].

In oligodendrocytes, genes related to the myelin sheath and paranodal junction assembly exhibited sex-dimorphic gene expression levels, and various genes involved in the formation of these two pivotal structures of oligodendrocytes were differentially expressed. For example, neurofascin gene (*Nfasc*) was significantly upregulated in males compared to females [26]. Myelin sheath was also affected in microglial cells, which control remyelination in the central nervous system (CNS) around the axons [27]. In heart endothelial cells, DE genes were widely involved in structural formation of the cardiopulmonary system. Three genes, *Ctnnb1*, *Notch1*, and *Nrp1* that regulate coronary artery morphogenesis were significantly upregulated in males. The, genes *Actb*, *Atp5b*, *Gnai2*, *Gnas*, *Kcna5*, *Kdr*, and *Myadm*, that are associated with the membrane raft were also upregulated in male heart endothelial cells [28, 29]. Given that the membrane raft of endothelial cells is essential for signal transduction through key signaling molecules and ion channels [30, 31], this may be a new source of additional regulation for sex dimorphism that requires further investigation.

In astrocytes, genes related to the tetraspanin-enriched microdomain, *Tspan9* and *Pdpn* (cancer-related gene directly interacting with tetraspanin CD9 [32]), had significantly higher expression levels in male cells; hyaluronic acid binding-related genes also showed significant sex-biased expression, including *Bcan* which was more expressed in males and *Ncan* which was more expressed in females. Differential expression of these genes suggests potential sex dimorphism in astrocyte activation and that could translate to differences in response to injury.

In heart-infiltrating leukocytes, in addition to DE genes annotated to immune-related terms such as MHC class I protein complex and T cell receptor binding, the GO term, Schaffer-collateral-CA1 synapse, was also shown to be influenced by differential gene expression, suggesting an interplay between heart, neurons in the CNS, and sex differentiation. Examples of these DE genes with increased expression were *Capzb* and *Srgn* in females, as well as *Actb* and *Ptpra* in males. However, though leukocytes infiltration is associated with neural development in CNS [33], the potential association between cardiac leukocytes and cardiac nervous system has not been fully elucidated. In cardiac smooth muscle cells, myosin II complex and actomyosin structure organization were influenced by sex-biased gene expression, which may directly contribute to sex dimorphism in cardiac function. The heavy-chain coding gene *Myh9* was upregulated in males while the light-chain coding gene *Myl9* was upregulated in females. *Trpm7*, a cation channel coding gene has been reported to be responsible for magnesium homeostasis in vascular smooth muscle [34], was more highly expressed in males. In addition, the cardiac troponin I coding gene *Tnni3* was more highly expressed in females, and this may reflect differential sensitivity to intracellular calcium flow during muscular force production [35].

It is possible that the unequal sample size in sex groups may influence the number of DE genes detected, and hence the thresholds used for statistical significance in our computational pipeline were set to be more stringent. Additionally, we noticed that when comparing the direction of the gene expression differences between male and female cells, one sex did not consistently dominate the other, across all cell types (Supplementary Figure 5). For example, brain endothelial cells had more DE genes that were upregulated in females (Supplementary Figure 5B), whereas in heart endothelial cells and fibroblasts (Fig. 2b and Supplementary Figure 5F), more DE genes were upregulated in males.

### Scalable variability in distribution indicates widespread sex-dimorphic regulation of gene expression.

The set of DD genes identified in each cell type varied tremendously and there were very few DD genes that were common across different types of cells (Supplementary Figure 6 and Supplementary Table 4). Many conserved DD genes were related to carcinogenesis in brain cells which may contribute to the sex-specific predisposition to various types of brain tumor, including those based on oncogenes *Nras* [36] and *Alkbh5* [37] and genes associated with tumor cell proliferation and/or metastasis in the brain such as *Spry2* [38] and *Cryab* [39, 40]. Additionally, *Tsix*, antagonist and repressor of *Xist*, was also a conserved DD gene and identified across four brain cell types.

Results of GSVA indicate that DD genes play an important role in sex dimorphism that complement the pathways enriched for DE genes, though the underlying regulatory mechanisms are less clear and interpretable. Based on word cloud analysis of four cell types in which the number of significantly differentially regulated gene sets exceeded 100, we found that many gene sets were associated with inter-cellular heterogeneity and hence could explain the variability observed in gene expression, such as differentiation, development, and morphogenesis (Supplementary Figure 7A-D). There were many cell type-specific functions that were also represented.

Many of the DD genes in fibroblasts were related to transport, such as the transmembrane transport of fibroblast growth factor-related hormones (e.g., catecholamine, dopamine), cholesterol and various ions (e.g., calcium, chloride, potassium, sodium). This result is potentially relevant to sex dimorphism with respect to the regulation of fibroblast growth, fibroblast cholesterol biosynthesis, and regulation and membrane potential induction or maintenance. In contrast, in both brain and heart endothelial cells, many differentially represented gene sets were involved in signaling pathways and metabolic pathways. For example, heart endothelial cells had sex-dimorphic representation of collagen metabolism, which could be related to the stimulating effect of endothelium on collagen synthesis of cardiac fibroblasts [41].

GSVA also revealed findings about transcriptional regulation underlying sex specificity and inter-cellular heterogeneity from a new aspect. For example, using the most significantly sex-dimorphic gene sets in fibroblast, we observed that cells in male mice generally showed stronger activities in pathways related to keratinization, epidermis development (Supplementary Figure 7E), etc. Differentially distributed expression of cytokeratin (*Krt17*) [42] and stratifin *Sfn* [43] were responsible for the differential representation of these two pathways, both of which had significantly different proportions of zeros and have been shown to play an essential role in the differentiation of keratinocytes and fibroblast-keratinocyte interaction.

It is noteworthy that a considerable proportion of cells collected from female mice had these pathways even more strongly represented than in male cells, though they did not necessarily resemble their male counterparts in the expression pattern of every pathway. We posited that for certain biological processes (e.g., keratinization) that are critical for fibroblasts in both females and males, some female cells might upregulate the expression of specific genes so that the sex-dimorphic effect can be mitigated. In female cells, activities of many pathways seemed to be stronger than in male cells. The pathway for “hormone activity” is one example, where the differential proportion of zeros might be responsible for sex-dimorphic fibroblast growth (Supplementary Figure 7E). For example, peptide YY (coded by *Pyy*) is a gene that had differential proportion of zeros; it belongs to the gene set “hormone activity” and this gene is known to stimulate proliferation and collagen production of cardiac fibroblasts [44].

### Deconvolution of differential distribution in gene expression to further explain cell-cell heterogeneity

We posited that DD genes could be used to identify new sub-cell types within cell populations. Previous cluster analysis and identification of cell types did not decompose inter-cell expression variance into variance caused by sex specificity and variance caused by cell type specificity. Intuitively, major cell types have distinct gene expression signatures that make them easily recognizable by canonical algorithms because the variance is mainly due to cell type specificity. However, when sex specificity is the predominant cause of the variance, identifying subpopulations of cell types is more challenging. We presume that DD genes would be able to account for the sex-specific variance without introducing new covariates. Then, further classification can focus on sub-cell type specificity and can be a promising approach to uncovering biologically meaningful cell groups.

**Fig. 3:**
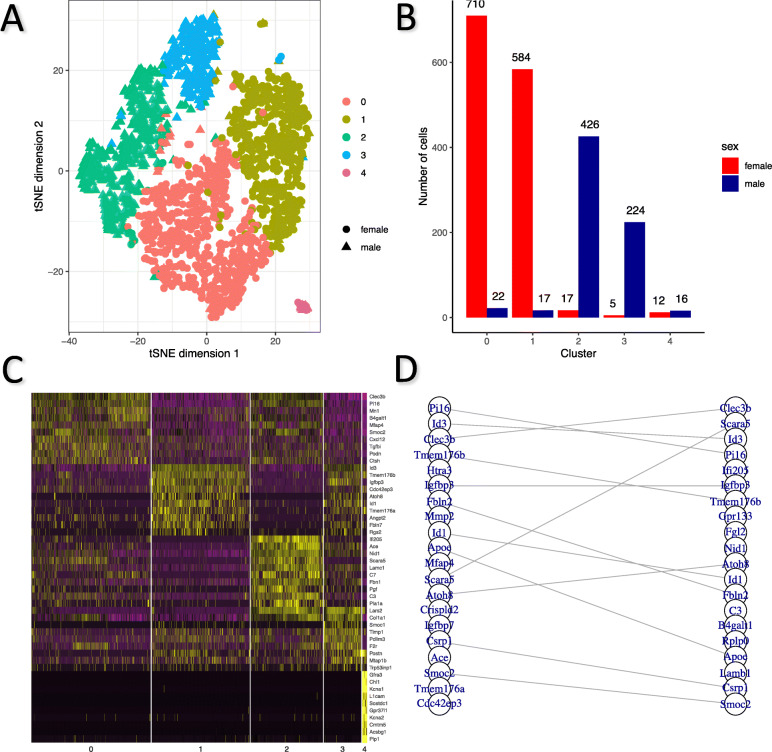
Sub-classification of fibroblast cells identified sub-cell types. **a** Five sub-cell types of heart fibroblast cells were identified by unsupervised clustering. Most female cells formed cluster 0 and 1 while most male cells formed cluster 2 and 3. **b** Distribution of male/female cells in each cluster. Cluster 0, 1, 2, and 3 exhibited dominance of one sex, while cluster 4 was a mixture of both sexes. **c** Expression of top ten marker genes distinguishing each cluster from the other clusters indicated strong sub-cell type specificity. Nonetheless, expression patterns were perceptually more similar between cluster 0 and 2 as well as those between cluster 1 and 3, than other pairs of clusters. Cluster 4 stood out as almost all corresponding marker genes were hardly expressed in other four clusters. **d** Top 20 marker genes distinguishing cluster 0 from 1 (left) and cluster 2 from 3 (right) showed a strong overlap. Twelve marker genes were conserved in top 20 most significant marker genes for both pairs of comparisons

We sub-classified fibroblast cells using DD genes as classifiers (the “Methods” section) and identified five cell clusters (Fig. 3a). Two clusters (0 and 1) were predominantly made up of female cells (97.0% of cluster 0 and 97.2% of cluster 1) whereas two clusters (2 and 3) were predominantly consisting of male cells (96.2% of cluster 2 and 97.8% of cluster 3; Fig. 3b). We found clusters 0 and 2 may have similar biological identities because the highly expressed unique marker genes of cluster 0 were moderately expressed in cluster 2 cells but not in other clusters, and vice versa (Fig. 3c). Likewise, clusters 1 and 3 also exhibited high similarity as determined by similar expression profiles of marker genes (Fig. 3c). Notably, one mixture cluster (cluster 4) of both female and male cells of comparable proportion exhibited different expression profiles compared to the other clusters. No evidence for differential expression of housekeeping genes and cell division or mitotic cell cycle-related genes was observed between female and male cells in clusters 0 and 2, clusters 1 and 3 and within cluster 4 (Supplementary Figure 8).

We then identified 238 marker genes that were differentially expressed between clusters 0 and 1, as well as 291 marker genes that were differentially expressed between clusters 2 and 3. Among the two lists of marker genes, 134 overlapped. These common marker genes were strongly associated with cellular components of extracellular space and basement membrane, and plasma membrane binding of crucial molecules, such as heparin, integrin, and calcium ion (Supplementary Table 5), which are essential for proliferation and cell-matrix interaction of fibroblast cells. Notably, marker genes that were most differentially expressed, as determined by FDRs, overlapped to a large extent (Fig. 3d), which suggests that the similar transcriptomic reshaping is associated with the diversification of cells in both females and males. In particular, *Clec3b* and *Pi16*, two genes known to be significantly upregulated in Col14a1 matrix fibroblasts uniquely [45], had both higher level of expression and larger proportion of actively expressing cells in cluster 0 and 2, suggesting similarity of these two clusters to Col14a1 matrix fibroblasts. In contrast, *Id3* [46], *Tmem176b* [47], and *Igfbp3* [48], three genes widely involved in cell proliferation and differentiation as well as cellular aging in fibroblasts, were significantly upregulated in cluster 1 and 3, implying that cells in these two clusters might be involved in a dynamic process of reproduction and differentiation.

Interestingly, though the marker genes for the distinct mixed-sex cluster 4 had exclusive and unique expression profiles, they were also associated with extracellular matrix/cell surface-related components and functions as mentioned above (Supplementary Table 6). This emphasizes the important role of signaling transduction, adhesion and cell-cell interaction in establishing cell heterogeneity of fibroblast. Cells in this cluster, with actively transcribed *Kcna6* and *Kcna1* (Supplementary Figure 9), coding for potassium voltage-gated channels Kv1.1 and Kv1.6, respectively, are highly likely to be responsible for the generation and regulation of voltage-dependent potassium currents in heart fibroblasts [49, 50]. Meanwhile, these cells expressed various proteins that are commonly found in neural/glial cells, including the artemin receptor component GFRA3, the neural cell adhesion molecule L1-like protein CHL1, and the solute carrier SLC35F1, suggesting a close relationship with the nervous system.

### Gene regulatory networks identify hidden differential regulatory mechanisms and cell heterogeneity for sex-based differences

To identify potential drivers of sex-dimorphic transcriptional regulation, we augmented a Jack-knife-based PANDA framework to generate 100 female-specific networks and 100 male-specific networks in each of the cell types analyzed in this study (the “Methods” section). For each of the four ensembles of brain-specific cell type networks, interactions between 318 TFs and 9673 genes were quantified as edge weights which were subjected to parametric statistical tests. In the six ensembles of heart-specific cell type networks, interactions between 317 TFs and 9722 genes were measured and examined in the same manner. We subsequently summarized differential TF-regulation from four aspects: (1) genes on sex-specific edges, (2) TFs on sex-specific edges, (3) TFs that show differential levels of activity overall, and (4) genes that are differentially targeted by the overall TF activity.

**Fig. 4:**
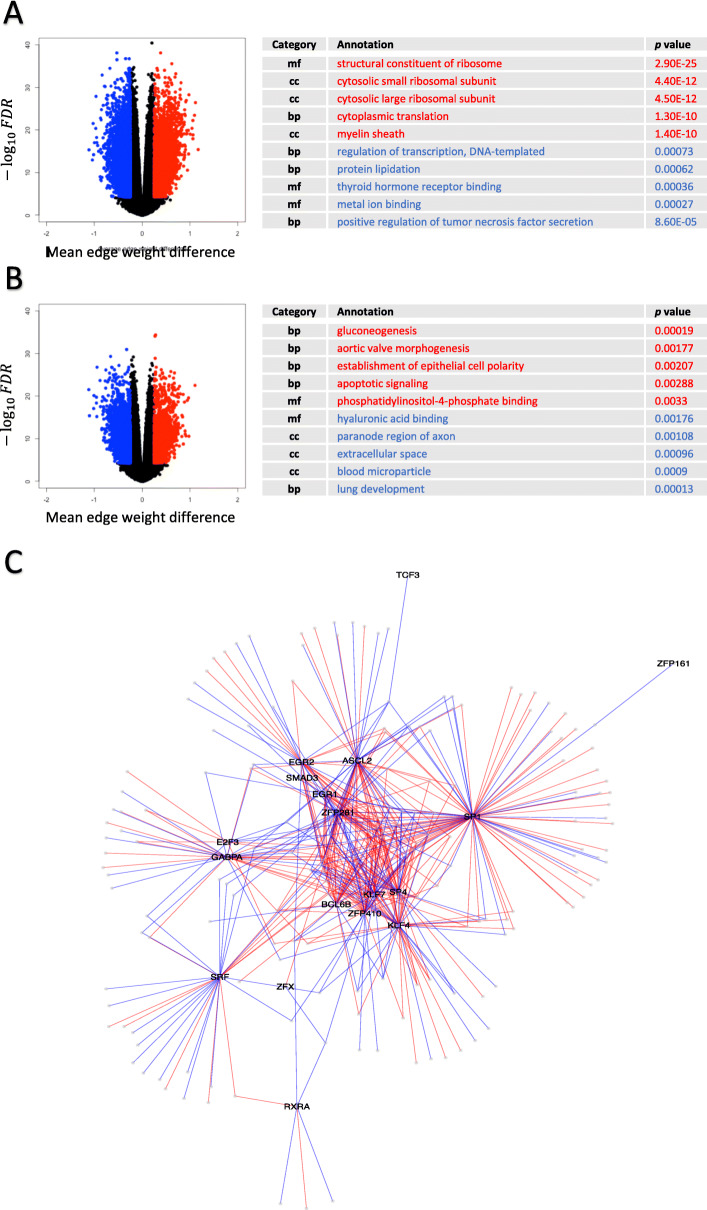
Differential edges influenced functional pathways in a cell type-specific manner. Volcano plots of edges in **a** cardiac muscle cells and **b** endocardial cells visualized differential edges with an FDR < 5 × 10^−5^ and a mean edge weight difference > 0.25 between females (red) and males (blue). Top five significantly enriched GO terms with smallest *p* values in female-specific edges (red letters) and male-specific edges (blue letters) were selected, respectively. Terms were ordered with regard to their statistical significance, female-specific term with the smallest *p* value at the top and male-specific term with the smallest *p* value at the bottom. **c** Network containing sex-specific edges in leukocytes was illustrated as it displayed moderate complexity suitable for visualization. Labeled nodes are TFs and unlabeled nodes are genes. Female-specific edges are colored red and male-specific edges are colored blue

While the number of network statistics, 100, used in each pair-wise Welch’s *t* test for differential edges was consistent, we observed dramatic changes in the number of significantly differentially represented edges across 10 cell types (Fig. 4a, b; and Supplementary Figure 10). Naturally, the number of genes and TFs involved in these differential edges also varied substantially. In most cases, genes on sex-specific edges did not show any overlap between females and males, namely most genes appeared exclusively in female-specific or male-specific edges, except for cardiac muscle cells where 487 genes were under both female-specific and male-specific regulatory control (Supplementary Table 7). The TFs responsible for sex-specific edges, on the other hand, showed strong overlap which seems plausible as a large proportion of TFs could be in control of both female-specific and male-specific edges simultaneously (Supplementary Table 7). Functional annotations of these genes that were differentially targeted by specific TFs provided further information underlying the regulation of tissue and cell type specificity (Supplementary Table 8 and Supplementary Table 9). Particularly, for cardiac muscle cells and endocardial cells where most genes were not differentially expressed, the genes on sex-specific edges were involved in many processes associated with specialized cellular functions. For example, in male cardiac muscle cells, genes related to positive regulation of tumor necrosis factor secretion were more strongly targeted by TFs than in female cardiac muscle cells (Fig. 4a). Sex-specific TF-gene binding pairs involved in this process included SP1-*Fzd5*, SP1-*Cyp2j6*, SP1-*C1qtnf4*, KLF4-*Tlr2*, NR5A2-*Cd84*, and ZFP281-*Arid5a*. In female endocardial cells, aortic valve morphogenesis is one of the processes that had stronger TF-targeting (Fig. 4b). Examples of sex-specific TF-gene binding pairs were SMAD3-*Efna1*, SP1-*Emilin1*, ZFP281-*Emilin1*, ASCL2-*Rb1*, SRF-*Rb1*, SP1-*Slit3*, SP1-*Smad6*, and ZFP281-*Smad6*.

**Fig. 5:**
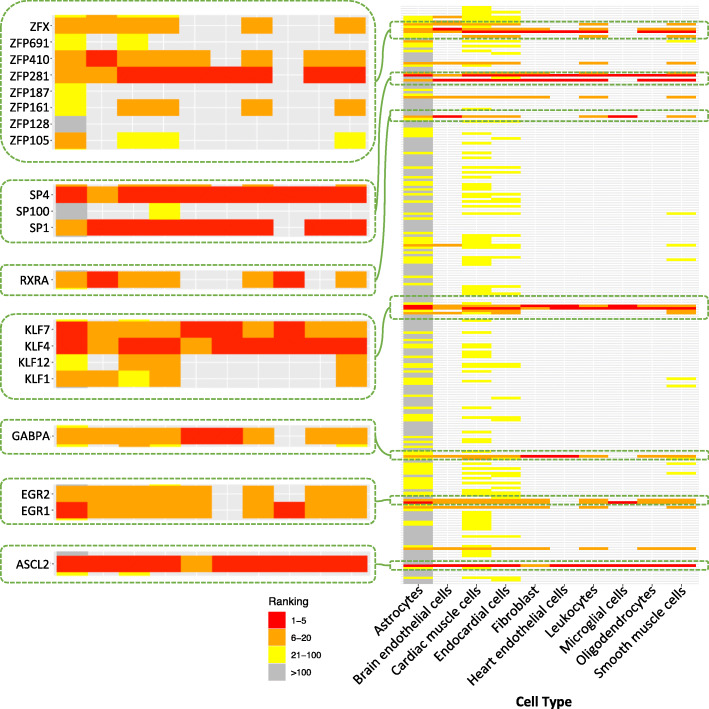
Core TF families were conserved in sex-specific regulatory TF-gene pairs. One row represents one TF. Light gray bins indicate that the corresponding TF was not involved in any sex-specific edge in the corresponding cell type. In each cell type, TFs were ranked based on the number of sex-specific edges from which they extend. The more sex-specific edges that a TF contributed to the PANDA network, the higher its ranking is. TFs were arranged in alphabetic order for illustration of TF families. TFs families containing member(s) ranked top 5 (colored red) in any one type of cell were viewed in zoomed-in windows. These TFs suggested seven core TF families showing remarkable property of conservation in all types of cells

While genes on differential edges exhibit high cell type specificity, TFs in control of the differential regulation through motif recognition and binding are highly conserved across cell types. From the illustration of sex-specific edges present in cardiac leukocytes (Fig. 4c), we observed several core nodes which are TFs with dense sex-specific edges, such as SP1, SP4, KLF4, ASCL2, and ZFP281. We further identified TFs that contribute the most to the differential edges by gathering information of all 10 types of cells under investigation (Fig. 5). These core TF families are ASCL2, EGR family (EGR1 and EGR2), GABPA, SP/KLF family (SP1, SP4, KLF4 and KLF7), RXRα, and members of the Zinc Finger family (ZFP281 and ZFP410). Despite the obvious variation in the number of differential edges and TF on differential edges, these TFs almost always initiate differential edges most frequently in the 10 cell types.

**Fig. 6:**
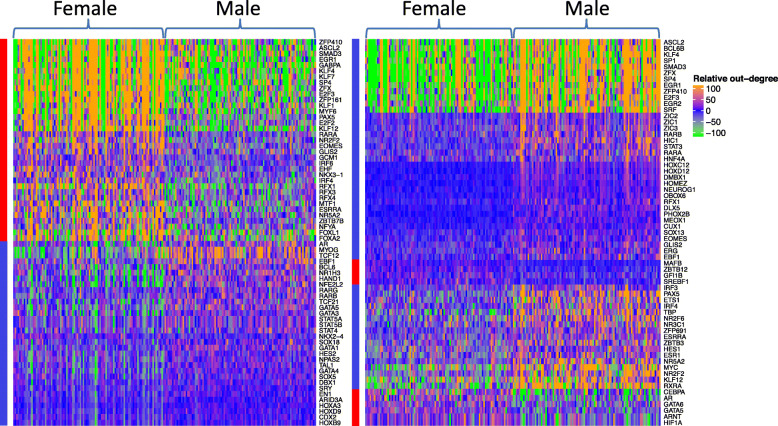
Significantly differential targeting TFs in astrocytes (left) and cardiac muscle cells (right). Each row represents one TF and each column represents one network. In each plot, 100 randomly generated female-specific networks were placed on the left half and 100 randomly generated male-specific networks were placed on the right without clustering. Relative out-degree values were obtained by subtracting median summed edge weight of each TF in all 200 networks of astrocytes/cardiac muscle cells from each individual summed edge weight. Rows were hierarchically clustered with distance measured by Pearson correlation. Bars mark TFs that were significantly more active in either females (red) or males (blue)

In addition, we identified TFs with differential overall activity which were quantified by summing the weights of edges extending from the same TF and illustrated these differentially targeting TFs using network ensembles of astrocytes and cardiac muscle cells (Fig. 6 and Supplementary Table 10). All TFs of previously identified TF families that accounted for the most differential edges also had differential overall activity in either of the two cell types shown, reinforcing their special role in relevant sex-dimorphic regulatory mechanisms. TFs belonging to the same TF family tended to have similar patterns in the intensity of activated regulation, as evidenced by the closeness of the unsupervised clusters. TFs are known to be universally responsible for sex determination and differentiation, and some TFs exhibited differential overall activity, including AR [51, 52], RAR [53], IRF [54], STAT [55, 56], and GATA [57]. Some TFs that have been known to be responsible for development in particular tissues were shown to have sex-dimorphic intensity of activity in the corresponding type of cell, such as MYC in cardiac muscle cells, which is able to regulate glucose metabolism and mitochondrial biogenesis [58, 59]. Meanwhile, TFs may also have opposite sex-specific activity in different cell types. For example, RARA had stronger overall activity in female astrocytes while also in male cardiac muscle cells.

**Fig. 7:**
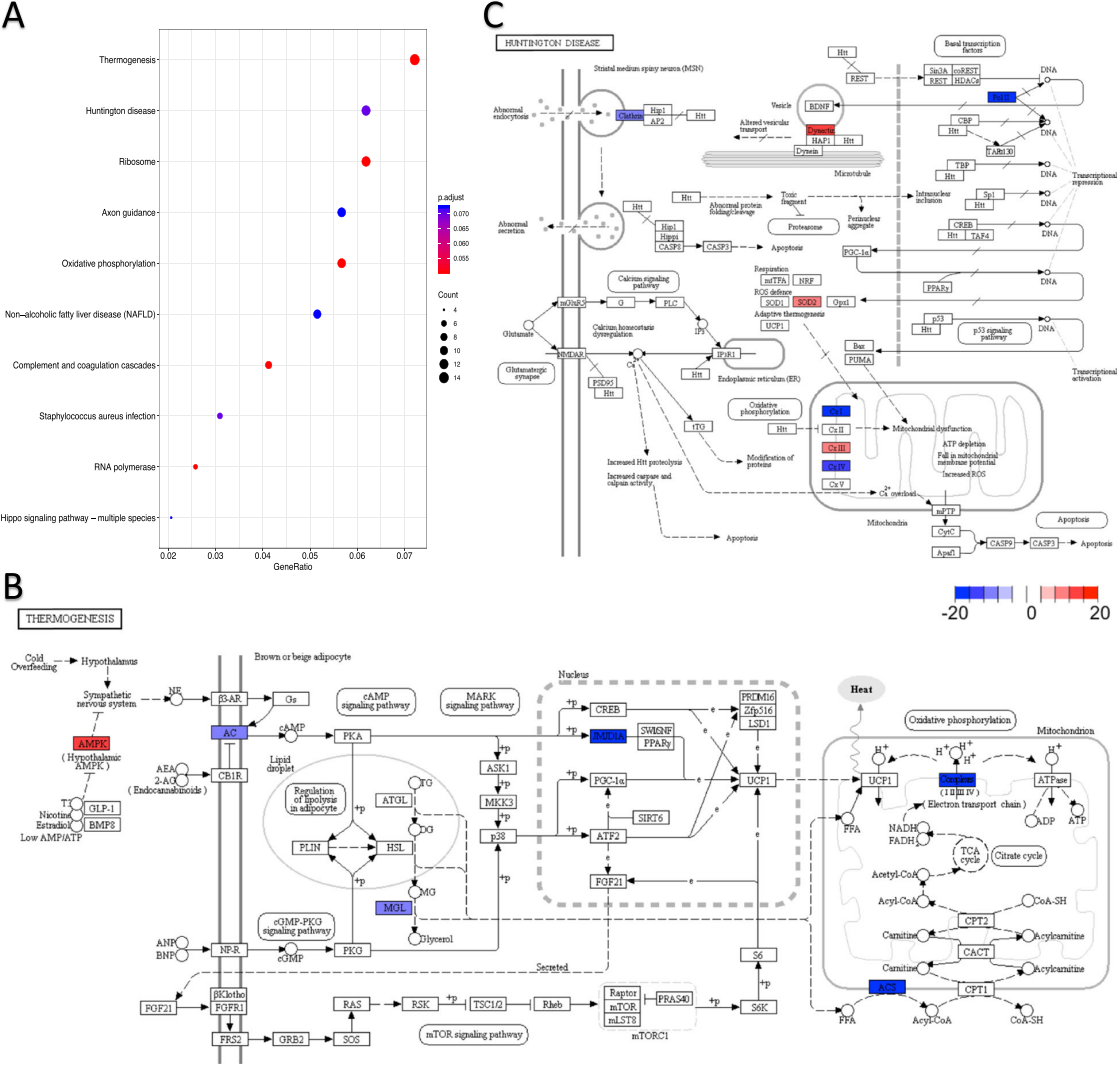
Differentially targeted genes in astrocytes were involved in both life-sustaining and disease-relevant pathways. **a** Bubble plot shows significantly enriched KEGG pathways (having at least five annotated genes) with an FDR < 0.1. Gene ratio stands for the proportion of differentially targeted genes in corresponding pathways. KEGG pathways of **b** thermogenesis and **c** Huntington disease are illustrated. Differentially targeted genes are colored red (female-specific) or blue (male-specific). Saturation is commensurate to the difference in summed edge weight

We also identified genes that were overall differentially targeted by summing weights of edges pointing towards the same gene. Due to the stringency of our threshold, only in astrocytes did we identify a considerable number of significantly differentially targeted genes (Supplementary Table 11). These genes were significantly enriched for KEGG pathways that stood out in analyses above such as ribosome, axon, and oxidative phosphorylation, as well as terms pertaining to neuronal activities in GO term enrichment analysis (Supplementary Table 12), but also thermogenesis and processes associated with Huntington’s disease which were the top two most enriched pathways (Fig. 7b, c). A broad range of key receptors and enzymes related to thermogenesis were under differential regulation, including exogenous hypothalamic AMPK, cell membrane enzymes adenylate cyclase AC and monoglyceride lipase MGL, and mitochondrial enzyme acetyl-CoA synthetase ACS and the electron transport chain complexes, as well as the nuclear enzyme JMJD1A, which is a H3 lysine 9 demethylase promoting expression of thermogenic genes during acute cold stress [60]. Dynactin, which is core to dynein/dynactin-mediated vesicle transport, was more strongly targeted in females, while Clathrin which is responsible for Clathrin-mediated endocytosis was more strongly targeted in males (Fig. 7c), together suggesting differential regulation of transport. Moreover, in both cardiac muscle cells and heart smooth muscle cells, the chemokine receptor Ccr5 which has been shown to be a key factor to atherogenesis in vascular smooth muscle cells [61], was more strongly targeted in females than in males (Supplementary Table 11). In both cardiac muscle cells and endocardial cells, I7Rn6 which is responsible for maintaining normal functioning of clara cells in lung development [62], was more strongly targeted in females than males as well. The cell adhesion molecule, NrCAM, though mainly expressed in brain and endocrine tissues, had the coding gene differentially targeted in cardiac leukocytes. These differentially targeted genes might imply connection in developmental process between tissues (e.g., blood vessels and cardiac cells).

Instinctively, we would expect genes on sex-specific edges and/or genes that are overall differentially targeted to have a significant overlap with DD genes since ideally TFs should change the expression pattern of their targeted genes. In this study, genes on sex-specific edges are more likely to be simultaneously differentially distributed for brain endothelial cells, cardiac muscle cells, endocardial cells, fibroblast, and leukocytes (Supplementary Table 13). However, being differentially distributed is independent of being overall differentially targeted in astrocytes (Supplementary Table 14).

## Discussion

Understanding baseline sex dimorphism at the molecular level is fundamental to assessing more complex sex-specific phenotypic differences, such as in disease pathogenesis, drug response, and life span. scRNA-seq provides opportunities to more accurately estimate cell-to-cell transcriptional variabilities and capture subtle cell type-specific differences compared to conventional bulk RNA sequencing. Leveraging Tabula Muris, a well-characterized large mouse single cell expression tissue atlas that yields high statistical power, we made extensive exploratory investigation into sex-dimorphic gene expression where DD and DE genes play an important role; we also identified key transcription factors that are potential drivers of sex-dimorphic transcriptional landscape.

In this study, DE and DD genes both demonstrate various functional importance. In addition to what has been demonstrated in cardiac fibroblasts, DE and DD genes are associated with diverse cell type-specific functional sex dimorphism (Supplementary Table 15), including but not limited to astrocyte-neuron symbiotic interaction, endothelial cell-regulated behaviors such as exploration and locomotion, and GABAergic neuron differentiation in microglial cells. Interestingly, we found more significant enrichment for DE genes and DD genes that are located on autosomes than on the sex chromosomes (Supplementary Table 16). This result was consistent with a recent study on sex-biased gene expression in adult human brain by analyzing different CNS regions [63]. We posit that differential expression is strongly cell type-specific and that difference may be undetectable for autosomal genes that are only differentially expressed in one or several types of cells when many existing studies investigated bulk samples. It is also possible that though sex-biased expression of genes on sex chromosomes may be prominent during early development, the central role of these genes in maintaining sex-specific differences may be dampened during adulthood. In contrast, the differential expression of autosomal genes may be the result of complex transcriptional regulation that permanently preserves sex-specific differences after puberty. It is also worth highlighting that the cutoffs imposed for differential expression were far more stringent than previous studies, and hence genes located on sex chromosomes that were identified as DE genes in earlier studies may have not reached statistical significance because of the higher degree of stringency in our study. Overall, our study has contributed substantial information to the understanding of widespread sex-dimorphic gene expression in the mouse using scRNA-seq data.

Our study has explored a new aspect of decomposing cell heterogeneity by first accounting for sex-dominating variance. Although we only selected fibroblasts for illustration because none of the DD genes in fibroblasts were uncategorized, the same method can be generalized to all cell types. Also, although we manually controlled the number of clusters being generated, more clusters can certainly be created as long as biologically meaningful interpretations are tenable. In addition to the comparison between female and male, DD genes can also be identified under other conditions (e.g., tumor cells and normal cells). This method can therefore be useful for explaining how these conditions influence the formation of subtypes of cells.

Our study also highlights the contribution of core TF families and their roles in sex-dimorphic gene expression regulation. These vital TFs include (i) ASCL2, a known controller of stemness in intestinal stem cells [64]; (ii) the EGR family, which are important for tissue plasticity and inducing cell-specific responses to proliferation, differentiation and apoptosis [65, 66]; (iii) GABPA, a necessary TF for mitochondria biogenesis and mitochondrial oxidation reactions [67]; (iv) KLF/SP family, a high-impact TF family that sustains a wide variety of biological processes including the maintenance of stemness, metabolism as well as differentiation [68]; (v) RXRα, which is associated with morphogenesis, heart development and cognitive abilities [69, 70]; and (vi) ZF family which is required for the realization of pluripotency [71]. It is noteworthy that all of these TFs are targets of and/or interact with the Wnt/β-catenin signaling pathway, one of the paramount pathways in sex determination and differentiation [72, 73]. Our analysis therefore suggests that these TFs are long-term executors of programmed sex dimorphism. Since gene expression is not necessarily regulated independently but instead interacts with and is controlled by other processes in the cell, we expect that sex-specific regulation of cell types may be further elucidated through inputs based on single cell DNA methylation and histone modifications.

Along with these findings, we have presented a novel computational pipeline for the analysis of transcriptional regulation underlying sex dimorphism at the single cell level for RNA-seq data. Our pipeline puts into effect the analysis of not only standard differential expression tests but also recently refined measurements of differential distribution that incorporate modality and quantify heterogeneity in gene expression directly. Our study has shown that gene expression profiles with differential distributions can be utilized in transcriptional processes that are tissue-specific and sex-specific through GSVA to unravel subtle changes in pathways. Importantly, this pipeline has extended the transcript-based gene regulatory network inference method, PANDA, to be applicable for scRNA-seq data, which improves the inference from the level of tissues to the level of identified cell types. Depending on the number of cells available, the statistically robust Jack-knife method used for constructing gene regulatory networks can also be generalized more broadly.

Our study has important limitations that are worth highlighting. First of all, for each organ, the Tabula Muris study sequenced several female and male mice. Though the mice used in the study were from an inbred strain and were housed under the same environmental conditions, it is indefinable how representative each mouse is of the general female or male mice population, because possible confounding effects of somatic mutations, stochastic noise introduced during rearing and sequencing, are elusive. Therefore, we cannot deny the risk of sampling bias in our study, which is, however, probably mitigated by the large number of cells, and we strongly recommend our results be considered exploratory and interpreted accordingly. It should also be noted that while each biological sample was collected from the same anatomical region from the female and male mice [14], heterogeneity may still exist even after cell sorting. It is likely that our study may still present results confounded to some degree by mixed sub-cell types as we described. We refrained from further profiling of identified sub-clusters due to gradually reduced statistical power. Furthermore, though the imputation method we adopted improved our data quality, no data-driven approach, to our knowledge, specifically accounts for various sources of potential bias arising from sex chromosome-related regulatory mechanisms, including dosage compensation [74]. While our primary findings focus on targets identified on autosomes, differentially expressed genes identified on the X chromosome must be cautiously verified. Nevertheless, we anticipate future studies with even larger sample sizes and higher cell type profiling resolution to overcome these obstacles.

## Perspectives and significance

In this study, we investigated sex-specific gene expression and transcriptional regulation from the single cell transcriptomes of ten cell types in mouse brain and heart. We made comprehensive observations on the widespread control of sex-based dimorphism as indicated by differences in the number of transcripts, the proportion of cell type groups carrying distinct functions, or their underlying regulatory potential as inferred through network models. These findings, combined with future experimental validations, are likely to provide a good source for understanding sex differentiation of the transcriptome, adjusting for sex-driven discrepancies in disease or case-control studies, as well as benchmarking transcriptomic studies of a large sample size.

## Supplementary information

**Additional file 1:.** Supplementary Tables

**Additional file 2:.** Table S2

**Additional file 3:.** Table S3

**Additional file 4:.** Table S9

**Additional file 5:.** Table S15

**Additional file 6:.** Supplementary Figures

## Data Availability

The scripts used for analysis during the current study and the datasets generated are available in the GitHub repository, https://github.com/tianyuan-lu/Tabula-Muris-Processing-single-cell-RNAseq.

## Abbreviations

CNS: Central nervous system
DB: Differential both
DD: Differential distribution
DE: Differential expression
DM: Differential modality
DP: Differential proportion
DZ: Differential zero proportion
FACS: Fluorescence-activated cell sorting
FDR: False discovery rate
GO: Gene Ontology
GSVA: Gene set variation analysis
HVG: Highly variable gene
KEGG: Kyoto Encyclopedia of Genes and Genomes
NC: Not categorized
PANDA: Passing Attributes between Networks for Data Assimilation
PC: Principal component
PCA: Principal component analysis
PWM: Position weight matrix
scRNA-seq: Single cell RNA sequencing
TF: Transcription factor
tSNE: t-distributed stochastic neighbor embedding
TSS: Transcription start site
ZFP: Zinc finger protein
