# Supplementary material for: Investigating transcriptome-wide sex dimorphism by multi-level analysis of single-cell RNA sequencing data in ten mouse cell types
Source: Biol Sex Differ. 2020 Nov 5;11:61. doi: 10.1186/s13293-020-00335-2 (PMC7643324; doi:10.1186/s13293-020-00335-2)
Supplement: Supplementary file 1 — Additional file 1:. Supplementary Tables [file 13293_2020_335_MOESM1_ESM.docx]

**Supplementary Tables**

**Investigating transcriptome-wide sex dimorphism by multi-level analysis of single cell RNA sequencing data in ten mouse cell types**

Tianyuan Lu and Jessica C. Mar

**Table S1. Summary of available cell types in brain and heart**

| Tissue | Cell type | Female | Male | Misc.* |
| --- | --- | --- | --- | --- |
| Brain | Astrocytes | 85 (32+30+23) | 307 (67+60+68+112) | 0 |
|  | Brain endothelial cells | 223 (76+86+61) | 390 (101+65+106+118) | 0 |
|  | Microglial cells | 2142 (565+795+782) | 2170 (478+485+536+671) | 0 |
|  | Oligodendrocytes | 114 (40+46+28) | 1059 (241+251+276+291) | 8 |
|  | Bergmann glial cells^$^ | 6 | 24 | NA |
|  | Brain pericyte^$^ | 28 | 104 | NA |
|  | Brain smooth muscle cells^$^ | 1 | 13 | NA |
|  | Macrophages^$^ | 12 | 24 | NA |
|  | Neurons^$^ | 26 | 170 | NA |
|  | Neuronal stem cells^$^ | 4 | 32 | NA |
|  | Oligodendrocyte precursor cells^$^ | 44 | 158 | NA |
| Heart | Cardiac muscle cells | 74 (22+30+22) | 63 (13+11+21+18) | 0 |
|  | Endocardial cells | 124 (39+52+33) | 49 (8+12+14+15) | 2 |
|  | Fibroblast | 1328 (381+484+463) | 705 (135+128+230+212) | 12 |
|  | Heart endothelial cells | 622 (181+279+162) | 494 (82+109+163+140) | 8 |
|  | Leukocytes | 181 (54+74+53) | 305 (55+59+96+95) | 2 |
|  | Smooth muscle cells | 69 (21+32+16) | 148 (28+30+54+36) | 0 |
|  | Erythrocyte^$^ | 5 | 6 | NA |

*Misclassification: number of cells showing inconsistent grouping with their corresponding labels

^$^: Cell types that were not used for downstream analyses

Number of cells in female/male individuals are number of cells retained for downstream analyses, decomposed into summation with respect to samples (corresponding to mouse ID in *Tabula Muris*: 3_38_F, 3_39_F, and 3_56_F for females; 3_8_M, 3_9_M, 3_10_M and 3_11_M for males).

**Table S2. Summary statistics of differential distribution analysis in ten cell types**

Additional File

**Table S3. Top ten enriched GO terms for DE genes in eight cell types**

Additional File

**Table S4. Distribution of DD genes in all cell types**

| Cell type | DE | DM | DP | DB | DZ | NC | Total |
| --- | --- | --- | --- | --- | --- | --- | --- |
| Astrocytes | 47 | 778 | 65 | 254 | 9 | 1643 | 15268 |
| Brain endothelial cells | 251 | 128 | 87 | 44 | 513 | 3 | 15268 |
| Microglial cells | 279 | 375 | 185 | 1116 | 3474 | 0 | 15268 |
| Oligodendrocytes | 148 | 114 | 18 | 28 | 171 | 4 | 15268 |
| Cardiac muscle cells | 11 | 50 | 37 | 9 | 0 | 63 | 15160 |
| Endocardial cells | 22 | 1055 | 218 | 177 | 164 | 1214 | 15160 |
| Fibroblast | 237 | 251 | 157 | 736 | 2047 | 0 | 15160 |
| Heart endothelial cells | 49 | 46 | 107 | 57 | 1015 | 0 | 15160 |
| Leukocytes | 47 | 960 | 147 | 418 | 348 | 1871 | 15160 |
| Smooth muscle cells | 42 | 964 | 262 | 395 | 257 | 1336 | 15160 |

Total numbers are genes under investigation in corresponding cell types. Brain cells and heart cells have different total numbers of genes since some low-quality genes did not overlap.

**Table S5. Top ten enriched GO terms for marker genes distinguishing cluster 0/2 from 1/3**

| Category | GO ID | Annotation | *p* value |
| --- | --- | --- | --- |
| cellular component | GO:0005615 | extracellular space | 1.30E-25 |
| cellular component | GO:0005604 | basement membrane | 3.20E-16 |
| biological process | GO:0030198 | extracellular matrix organization | 5.60E-13 |
| cellular component | GO:0031012 | extracellular matrix | 2.40E-10 |
| molecular function | GO:0008201 | heparin binding | 4.90E-10 |
| molecular function | GO:0005201 | extracellular matrix structural constituent | 5.80E-08 |
| biological process | GO:0007155 | cell adhesion | 1.00E-07 |
| molecular function | GO:0005178 | integrin binding | 2.80E-07 |
| molecular function | GO:0005509 | calcium ion binding | 9.00E-06 |
| cellular component | GO:0005614 | interstitial matrix | 1.10E-05 |

**Table S6. Top ten enriched GO terms for marker genes distinguishing cluster 4 from other cell clusters**

| Category | GO ID | Annotation | *p* value |
| --- | --- | --- | --- |
| cellular component | GO:0005615 | extracellular space | 6.70E-28 |
| cellular component | GO:0005604 | basement membrane | 2.70E-15 |
| cellular component | GO:0009986 | cell surface | 8.70E-11 |
| biological process | GO:0030198 | extracellular matrix organization | 1.10E-10 |
| cellular component | GO:0031012 | extracellular matrix | 2.50E-09 |
| molecular function | GO:0008201 | heparin binding | 2.60E-09 |
| cellular component | GO:0005614 | interstitial matrix | 2.10E-07 |
| molecular function | GO:0005201 | extracellular matrix structural constituent | 3.70E-06 |
| molecular function | GO:0005509 | calcium ion binding | 5.00E-06 |
| molecular function | GO:0005518 | collagen binding | 6.50E-06 |

**Table S7. Overlapping of genes and TFs on sex-specific edges in all cell types between female and male**

| Cell Type | Genes | | | TFs | | |
| --- | --- | --- | --- | --- | --- | --- |
|  | F* | O^$^ | M^#^ | F | O | M |
| Astrocytes | 935 | 35 | 1573 | 16 | 213 | 5 |
| Brain endothelial cells | 7 | 0 | 21 | 0 | 10 | 10 |
| Microglial cells | 1 | 0 | 2 | 1 | 0 | 5 |
| Oligodendrocytes | 10 | 0 | 6 | 1 | 5 | 5 |
| Cardiac muscle cells | 1433 | 487 | 592 | 9 | 40 | 56 |
| Endocardial cells | 654 | 6 | 882 | 21 | 31 | 8 |
| Fibroblast | 6 | 0 | 10 | 0 | 7 | 7 |
| Heart endothelial cells | 3 | 0 | 2 | 3 | 2 | 2 |
| Leukocytes | 96 | 0 | 84 | 1 | 15 | 2 |
| Smooth muscle cells | 546 | 3 | 569 | 3 | 23 | 3 |

*F: Number of genes/TFs only on female-specific edges

^$^O: Number of genes/TFs on both female-specific and male-specific edges

^#^M: Number of genes/TFs only on male-specific edges

**Table S8. Sex-specific edges in five cell types.**

| Cell type | Female-specific edges | | Male-specific edges | |
| --- | --- | --- | --- | --- |
|  | Number | Genes | Number | Genes |
| Brain Endothelial cells | 17 | 2410001C21Rik, Arl2, C2cd2, Grb14, Hnrnpa0, Mbnl2, Stx12 | 77 | Atp5e, Atp5j, Brd7, Cox8a, Gba, Nipa2, Nucb1, Pkig, Ppib, Rpl18a, Rpl31, Rpl32, Rpl35, Rpl9, Rplp1, Rps14, Rps17, Rps26, Rps27, Rps7, Uba52 |
| Microglial cells | 1 | Fcgr3 | 6 | Arglu1, Marcks |
| Oligodendrocytes | 16 | Cwc27, Dusp19, Fech, Galc, Mfsd6, Rab31, Spag9, Syt11, Thoc7, Tle1 | 17 | Apod, Atp5l, Eci2, Ly6a, Mrpl13, Timm13 |
| Fibroblast | 11 | B2m, Bcl6, Id3, Lars2, Serinc3, Tmem176b | 44 | Alkbh5, B4galt1, Cbx5, Dpysl2, Kdelr2, Pam, Pdpn, Pi16, Serpinh1, Slc39a1 |
| Heart endothelial cells | 7 | Lpl, Ramp2, Tns1 | 5 | Gm20594, Serpinh1 |

Sex-specific edges have an absolute mean edge weight difference > 0.25 as well as an FDR < $5\times{10}^{-5}$. One gene can be the node of multiple sex-specific edges.

**Table S9. Top ten enriched GO terms for genes on sex-specific edges in five cell types**

Additional File

**Table S10. Differentially targeting TFs in all cell types except astrocytes and cardiac muscle cells**

| Cell type | Female* | Male^$^ |
| --- | --- | --- |
| Brain Endothelial cells | CEBPA | ERG, NRSA2, PAX5, RARA, ZIC1 |
| Microglial cells | ETS1, GABPA |  |
| Oligodendrocytes | ASCL2, FOXL1, MTF1, MYB, ZFP161, ZFP410 |  |
| Endocardial cells |  | BCL6B, EBF1, ESR1, GLIS2, HBP1, KLF1, MYOD1, PLAGL1, RUNX1, SFPI1, ZBTB7B, ZFP281 |
| Fibroblast |  |  |
| Heart endothelial cells |  |  |
| Leukocytes | BHLHE40, MYCN, NR3C1 | STAT6 |
| Smooth muscle cells |  | GFI1B, KLF1, ZFP691 |

*Female: TFs being overall more active in females

^$^Male: TFs being overall more active in males

Significantly differentially targeting TFs have an absolute mean difference in summed edge weights > 10 as well as FDR < 0.05.

**Table S11. Differentially targeted genes in all cell types except astrocytes**

| Cell type | Female* | Male^$^ |
| --- | --- | --- |
| Brain Endothelial cells |  |  |
| Microglial cells |  |  |
| Oligodendrocytes |  |  |
| Cardiac muscle cells | Ccr5, Dazap2, l7Rn6 | Acadm, BC013529, C1qbp, Dld, Eif4g2, Etfa, Gyg, Hspd1, Myl7, Nbn, Rab14, Rps7, Sdha, Sec13, Spnb2, Tspan3 |
| Endocardial cells | l7Rn6 |  |
| Fibroblast |  |  |
| Heart endothelial cells |  |  |
| Leukocytes | Nrcam |  |
| Smooth muscle cells | 2610305D13Rik, Ccr5, Tex9, Zfp316 |  |

*Female: genes receiving overall stronger TF-binding in females

^$^Male: genes receiving overall stronger TF-binding in males

Significantly differentially targeted genes have an absolute mean difference in summed edge weights > 10 as well as an FDR < 0.05.

**Table S12. Top ten enriched GO terms for genes differentially targeted in astrocytes**

| Sex | Category | GO ID | Annotation | *p* value |
| --- | --- | --- | --- | --- |
| Female | biological process | GO:0008286 | insulin receptor signaling pathway | 0.00036 |
|  | cellular component | GO:0030173 | integral component of Golgi membrane | 0.0015 |
|  | cellular component | GO:0005925 | focal adhesion | 0.0018 |
|  | molecular function | GO:0008013 | beta-catenin binding | 0.002 |
|  | molecular function | GO:0005225 | volume-sensitive anion channel activity | 0.0037 |
| Male | molecular function | GO:0046983 | protein dimerization activity | 0.00224 |
|  | biological process | GO:0008045 | motor neuron axon guidance | 0.00205 |
|  | molecular function | GO:0004653 | polypeptide N-acetylgalactosaminyltransferase activity | 0.00203 |
|  | biological process | GO:0007411 | axon guidance | 0.00188 |
|  | molecular function | GO:0003755 | peptidyl-prolyl cis-trans isomerase activity | 0.00029 |

The first five GO terms are enriched in genes more strongly targeted in females, with *p* values arranged in increasing order. The last five GO terms are enriched in genes more strongly targeted in males, with *p* values arranged in decreasing order.

**Table S13. Overlapping between genes on differential edges and differentially distributed genes**

| Cell type | Distribution | Dedge* | Non-Dedge | $\chi^{2}$ test *p* value | Adjusted *p* |
| --- | --- | --- | --- | --- | --- |
| Astrocytes | DDG^$^ | 449 | 1,379 | 0.1353 | 0.1933 |
|  | Non-DDG | 2,063 | 5,782 |  |  |
|  | Total | 2,512 | 7,161 |  |  |
| Brain endothelial cells | DDG | 19 | 665 | <2.2E-16 | <2.2E-16 |
|  | Non-DDG | 9 | 8,980 |  |  |
|  | Total | 28 | 9,645 |  |  |
| Microglial cells | DDG | 2 | 3,492 | 0.6167 | 0.7709 |
|  | Non-DDG | 1 | 6,178 |  |  |
|  | Total | 3 | 9,670 |  |  |
| Oligodendrocytes | DDG | 1 | 311 | 1 | 1 |
|  | Non-DDG | 15 | 9,346 |  |  |
|  | Total | 16 | 9,657 |  |  |
| Cardiac muscle cells | DDG | 40 | 68 | 0.01325 | 0.03633 |
|  | Non-DDG | 2,503 | 7,111 |  |  |
|  | Total | 2,543 | 7,179 |  |  |
| Endocardial cells | DDG | 325 | 1,504 | 0.01453 | 0.03633 |
|  | Non-DDG | 1,217 | 6,676 |  |  |
|  | Total | 1,542 | 8,180 |  |  |
| Fibroblast | DDG | 13 | 2,277 | 2.63E-07 | 1.32E-06 |
|  | Non-DDG | 3 | 7,429 |  |  |
|  | Total | 16 | 9,706 |  |  |
| Heart endothelial cells | DDG | 2 | 828 | 0.08582 | 0.14303 |
|  | Non-DDG | 3 | 8,889 |  |  |
|  | Total | 5 | 9,717 |  |  |
| Leukocytes | DDG | 59 | 2,386 | 0.02177 | 0.04354 |
|  | Non-DDG | 121 | 7,156 |  |  |
|  | Total | 180 | 9,542 |  |  |
| Smooth muscle cells | DDG | 236 | 1,864 | 0.6997 | 0.7774 |
|  | Non-DDG | 882 | 6,740 |  |  |
|  | Total | 1,118 | 8,604 |  |  |

*Dedge: genes on sex-specific edges

^$^DDG: differentially distributed genes

Due to the complexity of differential distributions, DD genes were not further categorized. For four brain cell types and six heart cell types, only 9,673 and 9,722 genes used for PANDA network construction were considered, respectively. *p* values were generated with Yates’ continuity correction and were adjusted for multiple testing using Benjamini-Hochberg method.

**Table S14. Overlapping between differentially targeted genes and differentially distributed genes in astrocytes**

|  | DTG* | Non-DTG |
| --- | --- | --- |
| DDG^$^ | 103 | 2,206 |
| Non-DDG | 319 | 7,045 |
| Total | 422 | 9,251 |

*DTG: differentially targeted genes

^$^DDG: differentially distributed genes

Only 9,673 genes used for PANDA network construction were considered. $\chi^{2}$ test *p* = 0.8366 with Yates’ continuity correction.

**Table S15. Top differentially represented gene sets of (A) DD genes and (B) DE genes in all cell types**

Additional File

**Table S16. Chromosomal distribution of DE genes and DD genes**

| Chromosome | Genes under investigation | DE genes | DE percentage | DD genes | DD percentage |
| --- | --- | --- | --- | --- | --- |
| chr1 | 887 | 46 | 5.19 | 625 | 70.46 |
| chr2 | 1,163 | 67 | 5.76 | 841 | 72.31 |
| chr3 | 748 | 40 | 5.35 | 526 | 70.32 |
| chr4 | 883 | 55 | 6.23 | 643 | 72.82 |
| chr5 | 926 | 53 | 5.72 | 683 | 73.76 |
| chr6 | 755 | 38 | 5.03 | 538 | 71.26 |
| chr7 | 1,133 | 52 | 4.59 | 811 | 71.58 |
| chr8 | 760 | 42 | 5.53 | 565 | 74.34 |
| chr9 | 874 | 53 | 6.06 | 607 | 69.45 |
| chr10 | 718 | 44 | 6.13 | 506 | 70.47 |
| chr11 | 1,234 | 74 | 6.00 | 894 | 72.45 |
| chr12 | 506 | 17 | 3.36 | 367 | 72.53 |
| chr13 | 580 | 19 | 3.28 | 407 | 70.17 |
| chr14 | 519 | 23 | 4.43 | 363 | 69.94 |
| chr15 | 614 | 30 | 4.89 | 412 | 67.10 |
| chr16 | 461 | 26 | 5.64 | 332 | 72.02 |
| chr17 | 729 | 44 | 6.04 | 527 | 72.29 |
| chr18 | 409 | 19 | 4.65 | 277 | 67.73 |
| chr19 | 506 | 29 | 5.73 | 384 | 75.89 |
| chrX | 524 | 18 | 3.44 | 342 | 65.27 |
| Total | 14,929 | 789 | 5.29 | 10,650 | 71.34 |

For each chromosome, genes under investigation in brain and heart cells were merged, among which 14,933 were annotated to the mouse genome. DE/DD genes found in at least one type of cells were annotated to the mouse genome. Genes on chromosome Y were excluded in the preprocessing step.
