# Supplementary material for: Investigating transcriptome-wide sex dimorphism by multi-level analysis of single-cell RNA sequencing data in ten mouse cell types
Source: Biol Sex Differ. 2020 Nov 5;11:61. doi: 10.1186/s13293-020-00335-2 (PMC7643324; doi:10.1186/s13293-020-00335-2)
Supplement: Supplementary file 6 — Additional file 6:. Supplementary Figures [file 13293_2020_335_MOESM6_ESM.pdf]

Supplementary Figures

Investigating transcriptome-wide sex dimorphism by multi-level analysis of single cell RNA sequencing data in ten mouse cell types

Tianyuan Lu and Jessica C. Mar

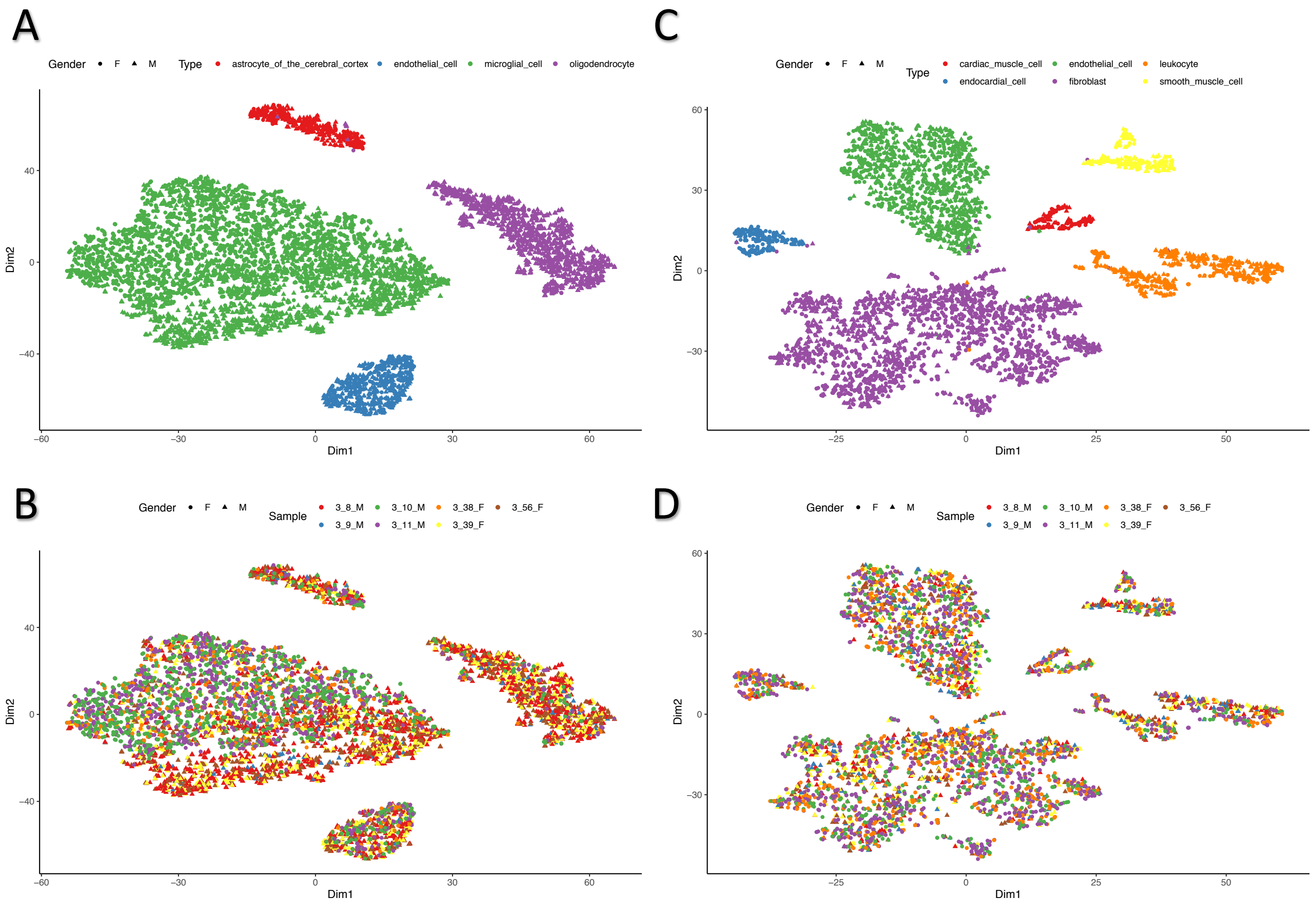

Figure S1. Representative tSNE clustering. tSNE clustering was illustrated for (A-B) 6,498 brain cells and (C-D) 4,186 heart cells. Cells were labelled using cell types (A) and (C) or mouse IDs (B) and (D) provided in the *Tabula Muris*. Most brain cells showed consistent grouping while eight oligodendrocytes appeared in the cluster of astrocytes of the cerebral cortex. Most heart cells also showed consistent grouping except for two endocardial cells, eight endothelial cells, 12 fibroblast cells and two leukocytes. Cells collected from different mice did not display overall discernible differences. Results were consistent in trials with five different random seeds. These aberrant cells were not included in downstream analyses. Summary statistics for distribution of cells in each cluster with respect to sex and sample ID are available in Supplementary Table 1. F: Female; M: Male.

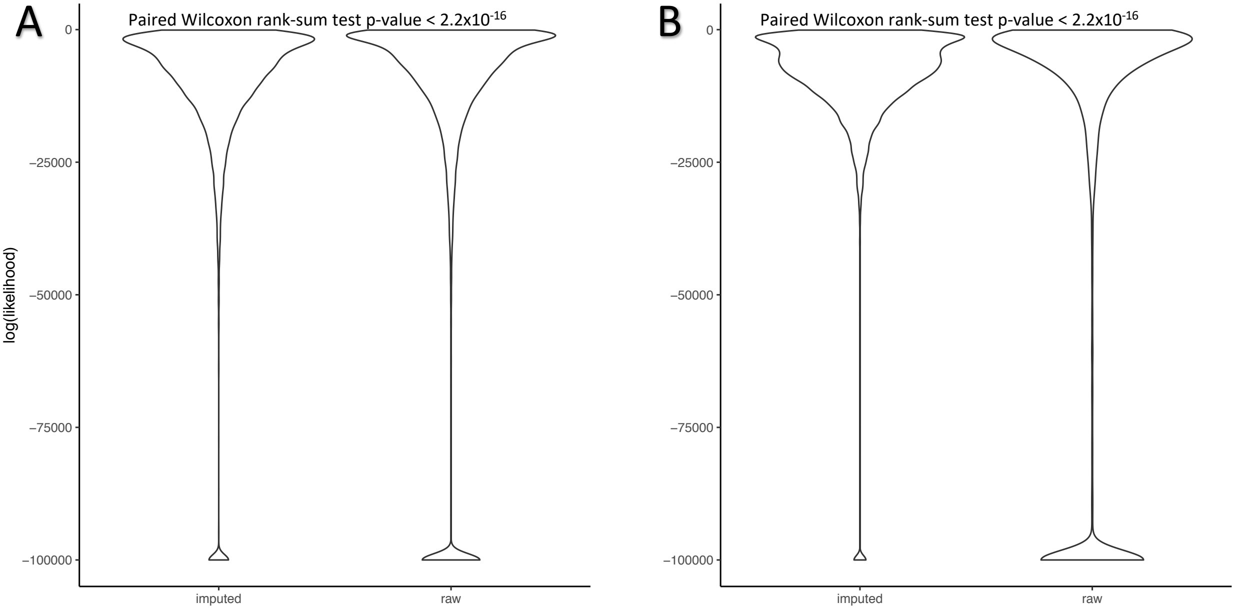

Figure S2. Summary of goodness-of-fit by negative binomial distribution. Negative binomial distribution was fitted based on each of (A) 15,268 available genes in brain cells and (B) 15,160 genes in heart cells. A high log-likelihood obtained from negative binomial model indicates strong similarity to a negative binomial distribution. Wilcoxon rank-sum test for paired gene-wise log-likelihood suggests significantly improved data quality of imputed data for both brain and heart cells.

A

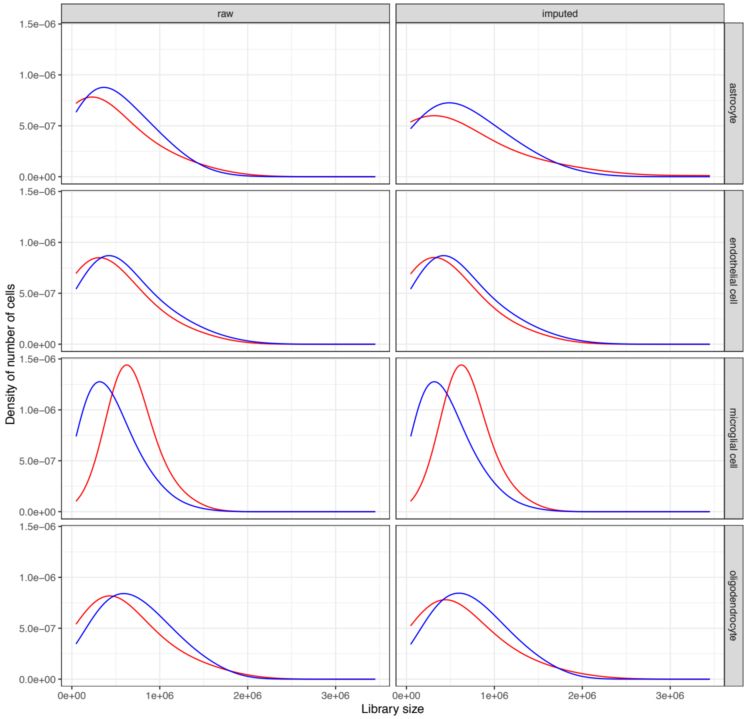

Median	Female	Male	Size Factor
raw	209,098.0	398,314.0	1.90
imputed	290,741.6	538,969.7	1.85

Median	Female	Male	Size Factor
raw	358,667.0	422,774.0	1.18
imputed	368,737.0	422,774.0	1.15

Median	Female	Male	Size Factor
raw	653,098.5	390,803.0	0.60
imputed	653,098.5	390,803.0	0.60

Median	Female	Male	Size Factor
raw	429,857.0	670,472.0	1.56
imputed	442,179.0	670,740.0	1.52

B

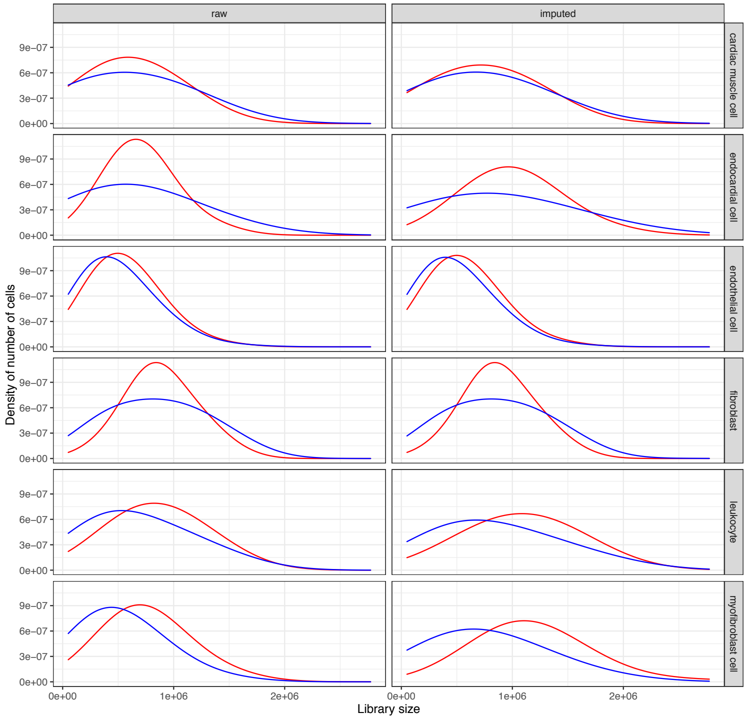

Median	Female	Male	Size Factor
raw	575,514.0	506,123.0	0.88
imputed	712,897.3	692,504.9	0.97

Median	Female	Male	Size Factor
raw	687,237.0	599,569.0	0.87
imputed	1,011,105.0	793,734.8	0.79

Median	Female	Male	Size Factor
raw	528,092.5	448,251.0	0.85
imputed	532,926.0	448,251.0	0.84

Median	Female	Male	Size Factor
raw	867,414.0	857,145.0	0.99
imputed	867,414.0	857,145.0	0.99

Median	Female	Male	Size Factor
raw	817,160.0	604,696.0	0.74
imputed	1,087,832.0	742,092.7	0.68

Median	Female	Male	Size Factor
raw	704,363.0	435,165.0	0.62
imputed	1,089,352.0	655,151.8	0.60

Figure S3. Distribution of library size before and after imputation. Individual library size for each female (red) and male (blue) cell is summarized for all cell types analyzed in (A) brain and (B) heart. Density curves were shifted to the right after imputation with consistent shapes. Changes in median library size also suggest that recovery of zero read counts in female and male cells was proportionate.

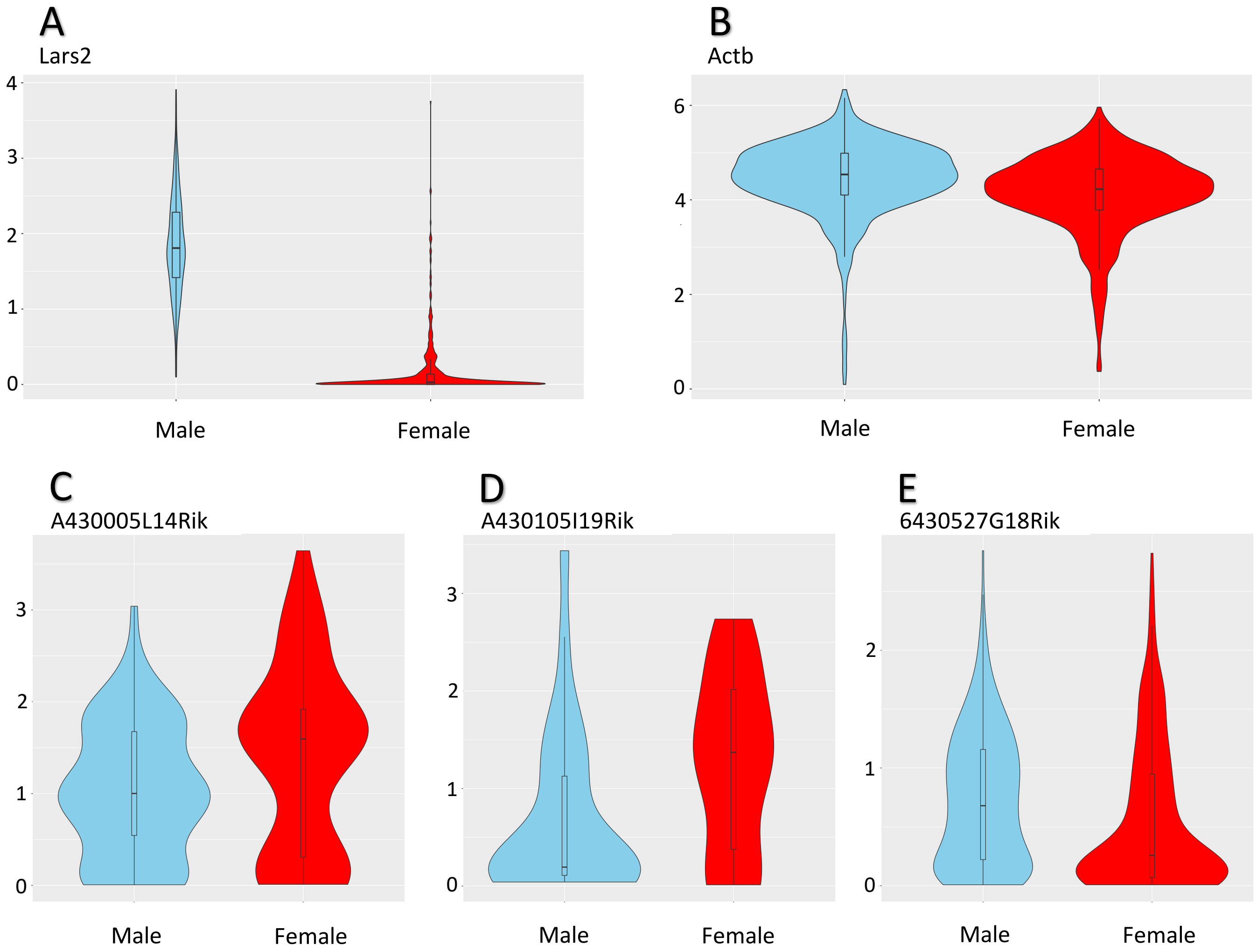

Figure S4. Examples of DD genes in heart endothelial cells. (A) Differential proportion of zeros and differential expression where most female cells did not express Lars2, (B) Differential expression where the expression level of Actb was significantly higher in male, (C) Differential modality where gene expression in male showed three modes while only two in female, (D) Differential proportion where gene expression showed two modes in both female and male but different proportion in each mode, and (E) Differential both differential modality and different component means where the expression level of 6430527G18Rik had two modes in male but only one in female, and neither expression mode in male overlapped with the expression mode in female. Expression values (y-axis) were imputed and normalized. Zero values were removed for (B), (C), (D) and (E) as scDD does not take into account zero values when testing for differential distributions.

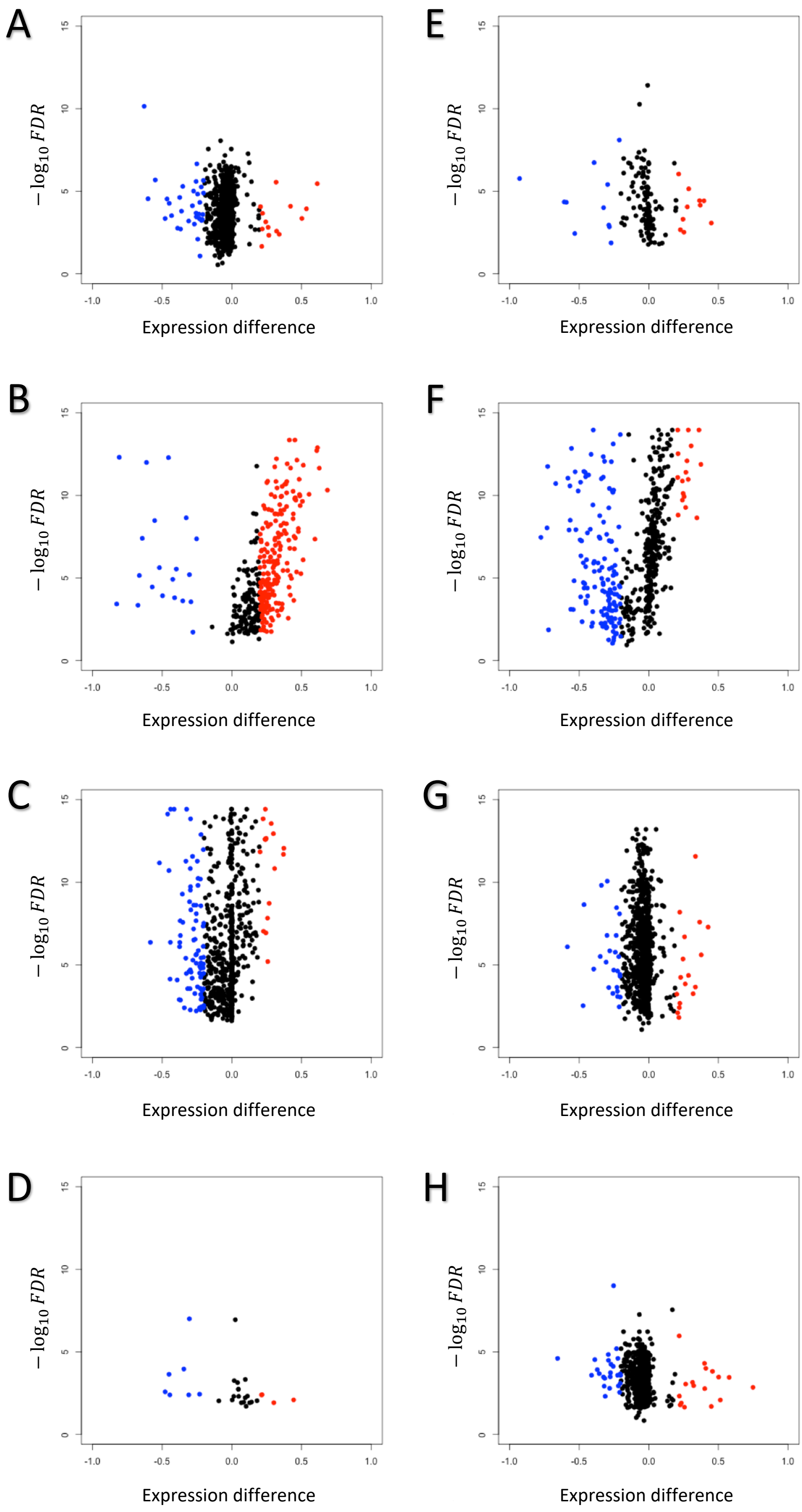

Figure S5. Volcano plots indicating cell-type specific differential expression patterns between female (red) and male (blue). Eight cell types were illustrated: (A) astrocytes, (B) brain endothelial cells, (C) microglial cells, (D) cardiac muscle cells, (E) endocardial cells, (F) fibroblast, (G) leukocytes and (H) smooth muscle cells. Expression differences were measured in log-normalized scale. Due to unequal sample sizes, tiny differences could gain strong statistical significance in large samples, resulting in potential false positivies. Thus, only genes with p values among the smallest 3% were regarded as DEGs in each cell type. Top 10 enriched GO annotations of these cell types are available in Supplementary Table 2.

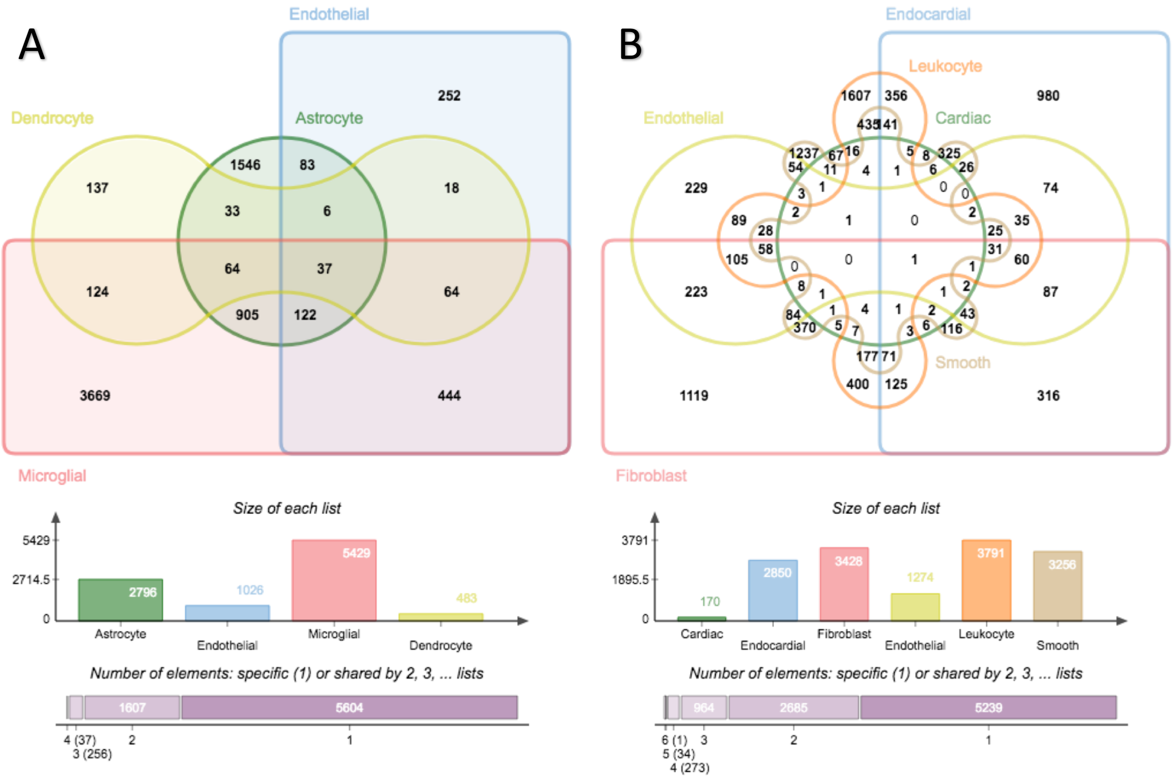

Figure S6. Cell type-specific DD genes exhibiting cellular heterogeneity. The number of DD genes varied in both (A) brain cell types (B) heart cell types. Only 37 in 7,504 brain DD genes were shared by all four brain cell types and only 1 in 9,196 heart DD genes was shared by all six heart cell types. ”Dendrocyte” in (A) represents oligodendrocyte; “Cardiac” in (B) represents cardiac muscle cell; “Smooth” in (B) represents smooth muscle cell.

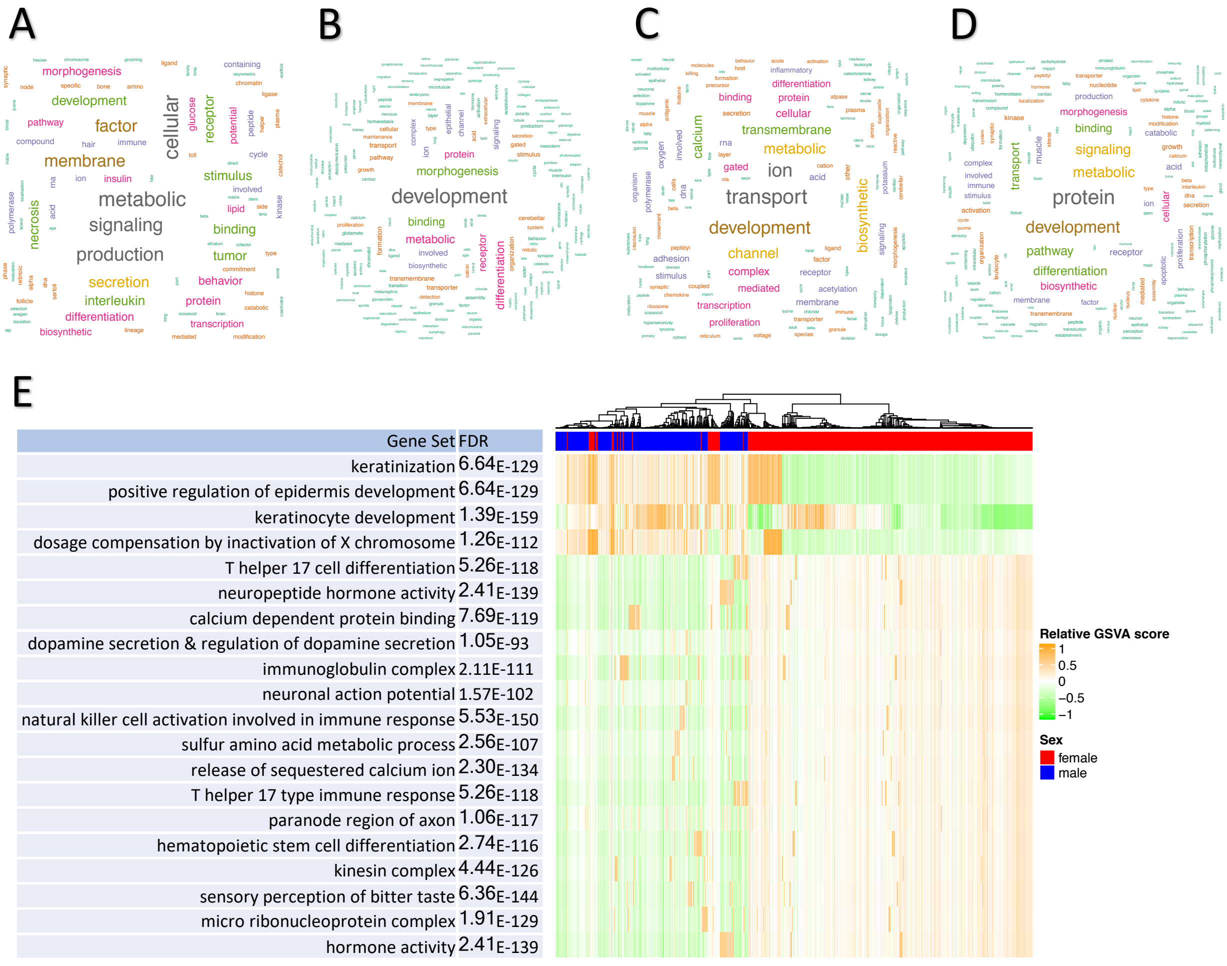

Figure S7. Gene set variation analysis identified pathways influenced by differential distribution of gene expression. Word clouds of keywords in differentially represented gene sets between females and males in (A) brain endothelial cells, (B) microglial cells, (C) fibroblast and (D) heart endothelial cells. More frequent keywords have larger fonts. (E) 20 gene sets with smallest FDR (after adjusting for multiple testing using Benjamini-Hochberg method) in fibroblast were visualized. GSVA scores were normalized to relative GSVA scores by subtracting mean GSVA score of each row. Rows matching gene sets on the left were arranged in numerically decreasing order according to mean differences calculated by mean GSVA score of males subtracting mean GSVA score of females. Each column represents one cell and was hierarchically clustered with distance measured by Pearson correlation.

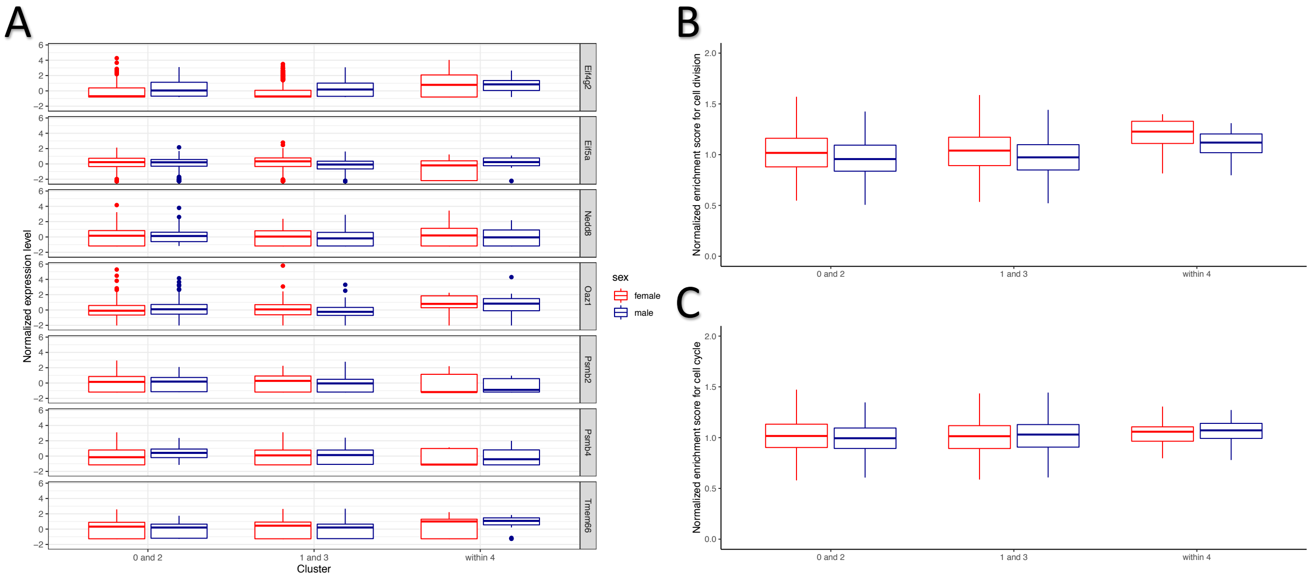

Figure S8. Housekeeping genes, cell division and cell cycle-related activities not involved in sex-dimorphic gene expression in cardiac fibroblast sub-clusters. (A) Exemplary housekeeping gene expression differences between female and male cells. Sub-cluster definition follows Figure 4. No evidence suggested housekeeping genes had sex-dimorphic expression (Benjamini-Hochberg-adjusted p-values for Wilcoxon rank-sum test > 0.05). Summary statistics for all 27 housekeeping genes are available in Supplementary Table 2. Gene set enrichment analysis revealed no significant difference in enrichment of (B) cell division or (C) cell cycle-related activities between female and male cells in each sub-cluster group (t-test p-values > 0.05).

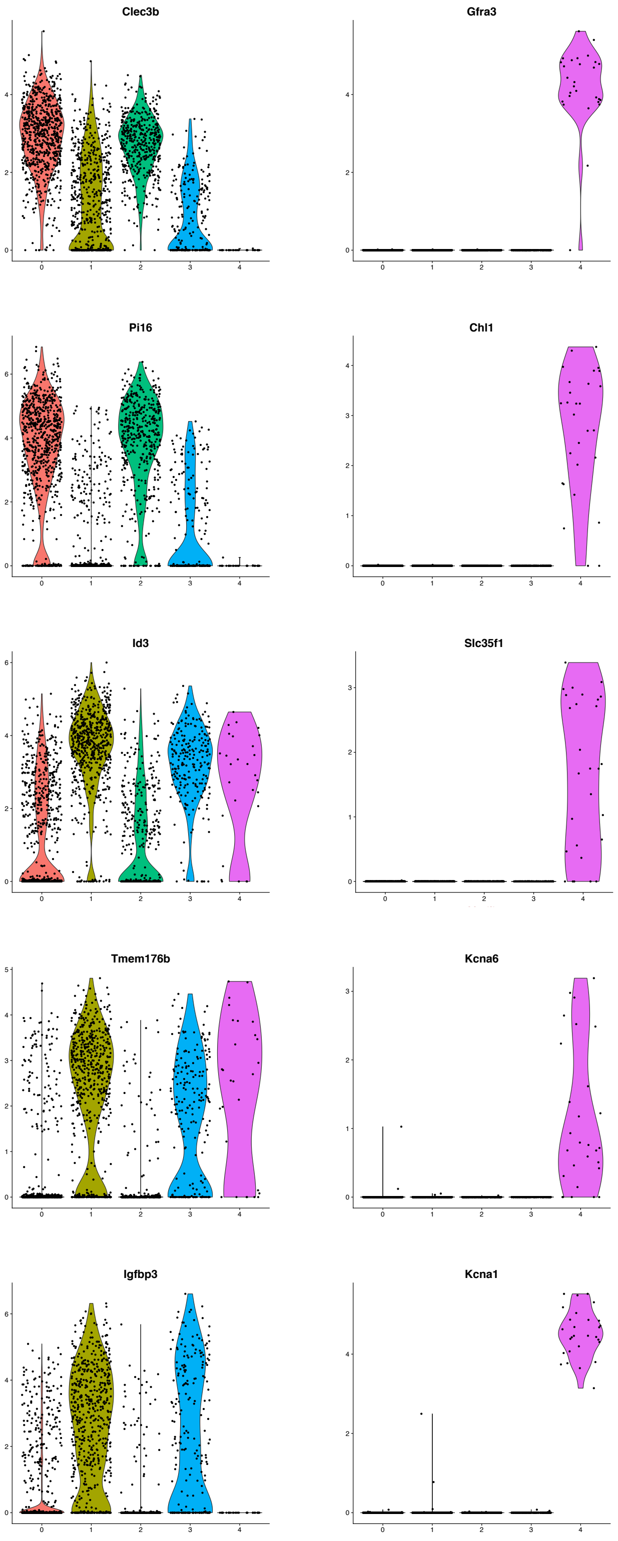

Figure S9. Expression profiles of marker genes in five sub-cell types of fibroblast. Clec3b, Pi16, Id3, Tmem176b and Igfbp3 were top five common marker genes distinguishing cluster 0 from cluster 1 as well as distinguishing cluster 2 from cluster 3 (Figure 3C). Top-ranked common marker genes were determined by summing rankings of marker genes in these two comparisons. Cluster 0 and cluster 2 had similar marker gene expression profiles while cluster 1 and cluster 3 had similar marker gene expression profiles. Gfra3, Chl1, Slc35f1, Kcna6 and Kcna1 were top five marker genes distinguishing cluster 4 from the other four clusters. These five genes were expressed almost exclusively in cluster 4. X-axis represents clusters and Y-axis represents normalized gene expression levels.

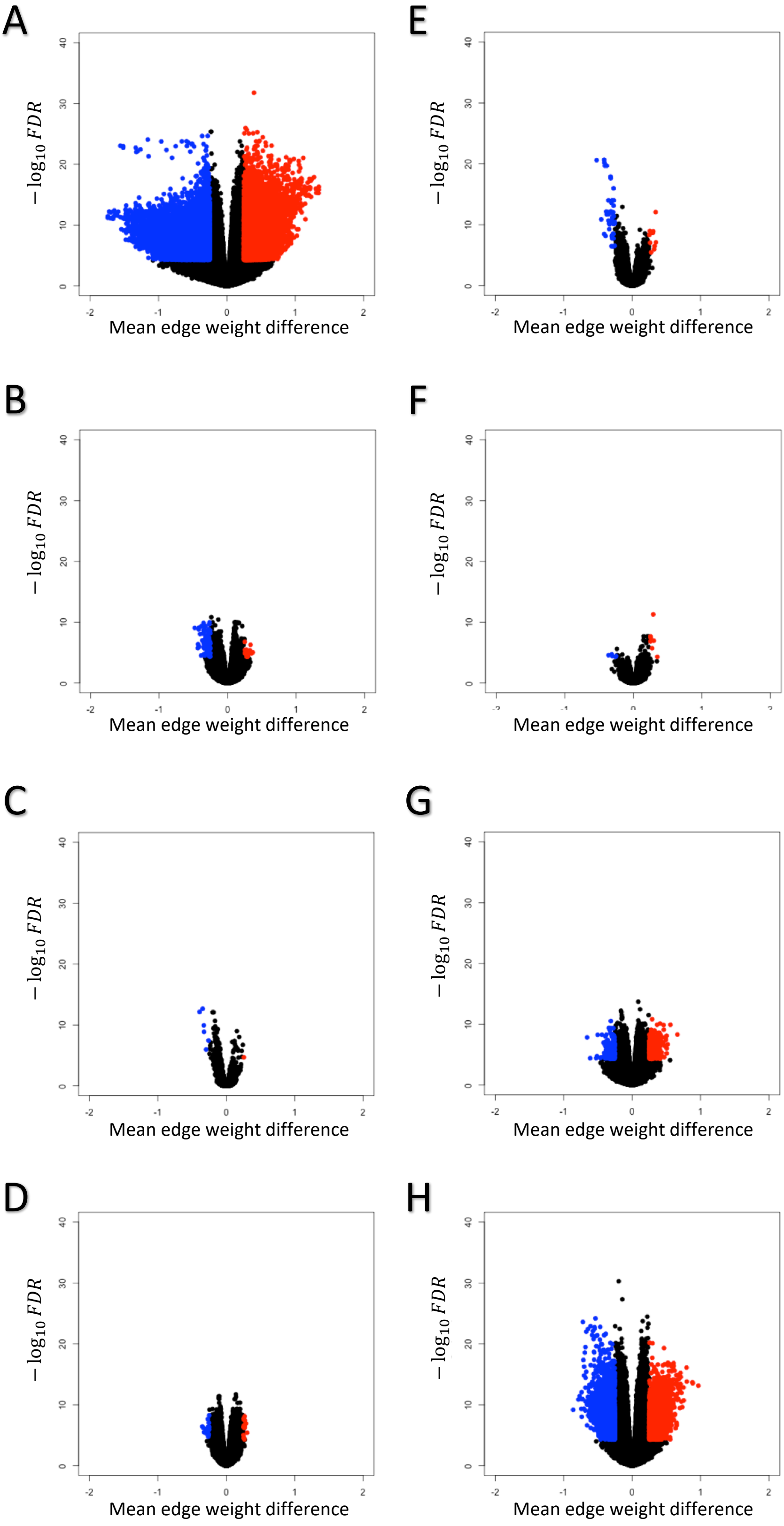

Figure S10. Volcano plots indicating cell type specific sex-specific edges. Eight (A) astrocytes, (B) brain endothelial cells, (C) microglial cells, (D) oligodendrocytes, (E) fibroblast, (F) heart endothelial cells, (G) leukocytes and (H) smooth muscle cells. Edges with adjusted Welch's t-test *p* value < 5×10^{-5} and absolute mean edge weight difference between female and male are colored to represent female-specific (red) and male-specific (blue) edges respectively. In astrocytes, leukocytes and smooth muscle cells, significantly differential edges were abundant (>100 genes involved in >100 edges). Top 10 enriched GO terms are available in Supplementary Table 8.
